# Illuminating Recent Progress in Nanotransfer Printing: Core Principles, Emerging Applications, and Future Perspectives

**DOI:** 10.1002/advs.202303704

**Published:** 2023-11-30

**Authors:** Junseong Ahn, Hanhwi Jang, Yongrok Jeong, Seongsu Choi, Jiwoo Ko, Soon Hyoung Hwang, Jun‐Ho Jeong, Yeon Sik Jung, Inkyu Park

**Affiliations:** ^1^ Department of Mechanical Engineering Korea Advanced Institute of Science and Technology (KAIST) Daejeon 34141 Republic of Korea; ^2^ Department of Nano Manufacturing Technology Korea Institute of Machinery and Materials (KIMM) Daejeon 34103 Republic of Korea; ^3^ Department of Materials Science and Engineering Korea Advanced Institute of Science and Technology (KAIST) Daejeon 34141 Republic of Korea; ^4^ Radioisotope Research Division Korea Atomic Energy Research Institute (KAERI) Daejeon 34057 Republic of Korea

**Keywords:** nanofabrication, nanoimprinting lithography, nanopatterning, nanotransfer printing, surface adhesion engineering

## Abstract

As the demand for diverse nanostructures in physical/chemical devices continues to rise, the development of nanotransfer printing (nTP) technology is receiving significant attention due to its exceptional throughput and ease of use. Over the past decade, researchers have attempted to enhance the diversity of materials and substrates used in transfer processes as well as to improve the resolution, reliability, and scalability of nTP. Recent research on nTP has made continuous progress, particularly using the control of the interfacial adhesion force between the donor mold, target material, and receiver substrate, and numerous practical nTP methods with niche applications have been demonstrated. This review article offers a comprehensive analysis of the chronological advancements in nTP technology and categorizes recent strategies targeted for high‐yield and versatile printing based on controlling the relative adhesion force depending on interfacial layers. In detail, the advantages and challenges of various nTP approaches are discussed based on their working mechanisms, and several promising solutions to improve morphological/material diversity are presented. Furthermore, this review provides a summary of potential applications of nanostructured devices, along with perspectives on the outlook and remaining challenges, which are expected to facilitate the continued progress of nTP technology and to inspire future innovations.

## Graphical abstract

The review highlights the increasing demand for diverse nanostructures in physical and chemical devices, leading to significant attention on nanotransfer printing (nTP) technology. The review categorizes and discusses various nTP approaches, their advantages, challenges, and potential solutions for improving morphological/material diversity. It also provides insights into potential applications of nanostructured devices and future outlook, encouraging further advancements in nTP technology.

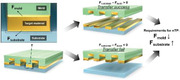

## 1 Introduction

Given the broad range of potential applications in the fields of physical, nanophotonic, and chemical devices such as physical/chemical/biological sensors,^[^ ^1^, ^2^, ^3^ ^]^ smart lenses,^[^ ^4^ ^]^ and displays,^[^ ^5^ ^]^ various nanofabrication technologies have garnered significant attention, while some of the technologies have subsequently become obsolete due to technical limitations. Nanofabrication processes can be broadly categorized into serial and parallel processes depending on their structure formation mechanisms. Serial processes (e.g., multiphoton lithography, focused ion beam lithography, and e‐beam lithography) are mainly based on point‐by‐point pattern formation using photon/electron‐sensitive materials or mechanical/chemical etching by incident ions.^[^ ^6^, ^7^ ^]^ Despite offering a broad spectrum of design options, the implementation of these methods is hindered by the need for costly fabrication equipment and low throughput, which poses a significant barrier to their commercialization. Therefore, to overcome these limitations, various parallel processes (e.g., holographic lithography, phase‐mask lithography, nanoimprinting lithography (NIL), nanotransfer printing (nTP), block copolymer (BCP), or DNA‐based 2D/3D self‐assembly) have been developed to provide nanostructures with high‐throughput and low‐cost processes.^[^ ^6^, ^7^ ^]^

Among them, nTP has attracted considerable attention in the industrial/academic fields because it has the high morphological/material diversity of printable materials and substrates, has high spatial resolution with reliability, and in particular, can be used to fabricate rationally designed nanostructures in large‐area with high‐throughput.^[^ ^8^, ^9^ ^]^ Chronologically, in the 2000s, Rogers et al. developed and led the advances of nTP techniques based on elastomer inking/stamping^[^ ^10^, ^11^, ^12^ ^]^ or thiol‐Au bonding.^[^ ^13^, ^14^ ^]^ Building upon this remarkable technological revolution, researchers have attempted to overcome bottleneck challenges, such as defectivity arising from the easy deformation of the elastomer mold or limited material diversity due to the use of thiol‐Au bonding. Since the 2010s, versatile nTP techniques have been developed by strategically controlling the interfacial adhesion force and using more reliable nanopatterned molds.

**Figure 1:**
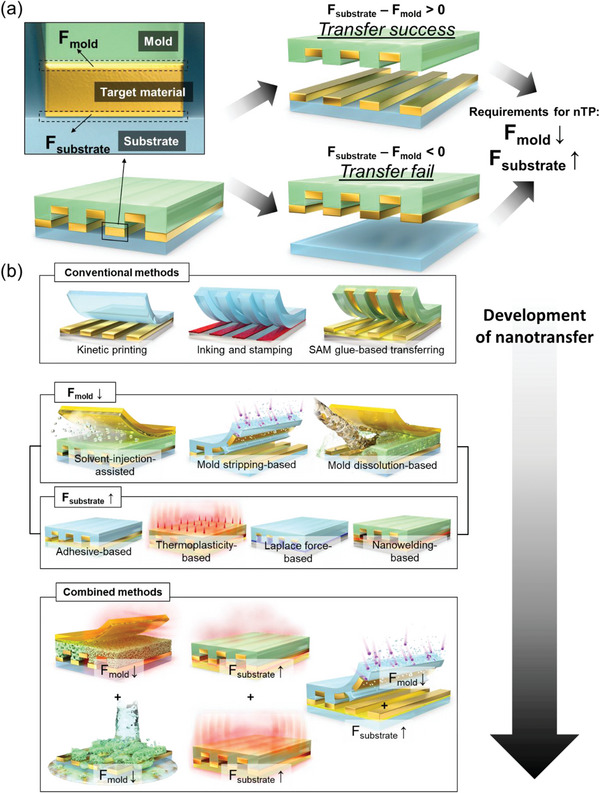
The overall concept of the nanotransfer printing technology. a) Schematic illustration showing the basic mechanism of nTP. b) Schematic illustration showing nTP's advancement from conventional nTP to combined adhesion control methods chronologically.

The basic mechanism of nTP can be explained by the relative interfacial adhesion force between mold, target material, and substrate, as shown in **Figure** **1**a. When *F_mold_*, the adhesion force between the mold and target material, is weaker than *F_substrate_*, the adhesion force between the target material and substrate (i.e., *F*_substrate_ − *F*_mold_ > 0), the nanopatterned target material can be transferred to the substrate. Therefore, two representative strategies for facilitating nTP have been exploited: i) *F*_mold_ weakening and ii) *F*_substrate_ strengthening. An example of the *F*_mold_ weakening method is the interpenetration of solvent vapor through the polymer chains of the mold that can act as a lubricant and reduce the *F*_mold_.^[^ ^8^ ^]^ In another example, one can directly strip the polymer mold to weaken the *F*_mold_ up to zero.^[^ ^4^, ^15^ ^]^ In contrast, as *F*_substrate_ strengthening strategies, mechanical interlocking through the deformation of thermoplastic substrate^[^ ^16^ ^]^ or metallic bonding through low‐temperature nanowelding^[^ ^17^ ^]^ can be generated between the target material and substrate. In another way, a commonly used adhesive was coated on the substrate to strengthen the *F*_substrate_ before performing nTP.^[^ ^18^ ^]^ Since the late 2010s, nTP based on the multi‐step and combined processes has been actively studied with progress being made in individual mechanisms for *F*_mold_ weakening or *F*_substrate_ strengthening. Combining two or more strategies simultaneously, such as the combination of mold stripping and adhesive coating, allows for the synergistic utilization of the advantages of each method, which enables easier release from the mold and stable printing on the substrate, respectively.^[^ ^19^ ^]^ Overall, recent nTP based on adhesion force control is being advanced toward improving the morphological/material diversity of the target materials and substrates with reliable large‐area processes. Despite the active progress in nTP research, a comprehensive and in‐depth review of the state‐of‐the‐art studies has yet to be conducted.

This review aims to fill this gap by summarizing the recent advances in nTP technology and categorizing them based on their working mechanisms, specifically focusing on the control of adhesion force, as shown in **Tables** **1** and **2**. In Section 2, we begin with the conventional nTP methods (e.g., elastomer‐based stamping, inking, Au‐thiol‐based bonding) in order to provide the basis for their evolution into the recent nTP methods. Then, Section 3 describes more recent nTP methods, from the representative fabrication of the nanopatterned mold to the detailed nTP mechanisms along with their advantages and limitations. As mentioned above, this review categorizes the fundamental mechanism of recent nTP into three distinct groups based on the relevant interfaces and transfer processes as follows: i) *F*_mold_ weakening strategies (e.g., solvent‐injection‐assisted and mold stripping‐based adhesion weakening), ii) *F*_substrate_ strengthening strategies (e.g., general adhesive‐based, thermoplasticity‐based, surface tension‐based, and nanowelding‐based adhesion strengthening), and iii) their combined processes. Various recent strategies that have been developed to overcome existing limitations are discussed, with a focus on the appropriate target materials and substrates for each method. Section 4 provides a summary of the emerging applications of nTP methods in physical, nanophotonic, and chemical devices, thereby confirming the necessity and unique advantages of nTP as a nanofabrication technology. Finally, Sections 5 and 6 address the current challenges and outline expected future research directions for the field of nTP.

**Table 1:** The summary and classification of nTPs depend on the main strategies for controlling the interfacial adhesion force between mold, target material, and substrate.

Main strategies for nTP			
Classification	*F*_mold_ weakening	*F*_substrate_ strengthening	Characteristics
*F*_mold_ weakening (Figure 4)	Solvent‐injection‐assisted super lubrication	‐	Facile transfer of the target materials on arbitrary surfaces without surface treatment, sequential printing for 3D stacked nanostructures, relatively unaffected from excessive heat and pressure
	Mold stripping by plasma etching	‐	
	Mold dissolution using solvents	‐	
*F*_substrate_ strengthening (Figure 5)	‐	Additional coating of adhesive	Generally, increase in stability of the nanotransfer printing itself and the final device Specifically, transferring depth control for the thermoplasticity‐based method, organic nanowire compatibility for Laplace force‐based method, and multi‐layer transferrable and superior electrical contact for the nanowelding‐based method
	‐	Enabling the conformal contact by thermoplastic deformation of the substrate	
	‐	Laplace force by polar liquid	
	‐	Nanowelding phenomena between metals	
Combined methods (Figure 6)	1) Thermal shrinkage of micropores in mold 2) Mold stripping by wet etching	‐	High morphological/material diversity of the target materials and substrates, high reliability in large‐area
	‐	1) Imidization‐induced mechanical interlocking 2) Thermoplasticity	High morphological diversity of the target pattern, including 3D shapes, high robustness
	Mold stripping by plasma etching	Adhesive	High morphological/material diversity of the target materials and substrates, high reliability in large‐area
	Mold stripping by wet etching	Adhesive	High morphological diversity of the substrate, such as nTP on a micropatterned substrate, high robustness

**Table 2:** Representative examples of recently reported nTPs, including their transfer conditions, applicable materials and substrates, and applications.

Reference	Main strategies for nTP	Required conditions for nTP	Applicable target materials	Applicable target morphologies	Applicable substrates	Applications
[68]	F_mold_ weakening (solvent‐injection‐assisted super lubrication)	Swelling PMMA sacrificial layer with warm (45°C) acetone/heptane co‐solvent vapor for 30 s.	Ag and Au	Nanowires, head‐to‐tail nanorods	Si wafer, flexible soft contact lens	Surface‐enhanced Raman spectroscopy
[15]	F_mold_ weakening (Mold stripping by water‐soluble polymeric molds)	Dissolving HA with a continuous supply of water	Pd, Ag, Al, SiO_2_	Line, dot, mesh, nanowires	Polyester, spandex, nylon, cellulose	Gas sensor, optical security label
[84]	F_mold_ weakening (Mold stripping by the orthogonal solvent)	Dissolving PMMA with acetone at room temperature for 35 min	CdSe, CdS, and ZnS quantum dots	Film, line, pixel, single particle	Si, PDMS, PTFE, curved vial surface, toothbrush fibers, fruit skin, leather, animal skin	Full‐color QLED
[18]	F_substrate_ strengthening (Adhesive‐based)	Coating of APS‐1 before nTP (spin coating with 5000 rpm, 1 min, baking with 150°C, 1min)	SiO_2_, Ag, Au, Al	Line (simple, rabbit ear‐shaped, Y‐shaped, asymmetric, hole‐shaped)	Si, glass, Al	Metasurface‐based hologram, wire grid polarizer
[32]	F_substrate_ strengthening (Thermoplasticity‐based)	90 ∼ 130 °C, 1 ∼ 7 bar, 1 ∼ 10 min of hot pressing	Ag, Au	Line, hole, dot, mesh, hologram surface	PMMA	Metasurface‐based ring hologram, color filter
[92]	F_substrate_ strengthening (Laplace force‐based)	Dry‐fabricated organic nanowire @ low‐surface energy nanomold, spin coating of polar liquid over the substrate	TIPS‐PEN, C_60_, P3HT	Line	Poly(ether sulfone)	Organic FET, inverter, and p‐n diode arrays
[17, 61, 67]	F_substrate_ strengthening (Nanowelding‐based)	90 ∼ 190 °C with a thickness of target material in the range of 20 ∼ 50 nm	Ag, Au, Pd, Pt	Line, dot, mesh	Ag, Au, Pd, Pt	Gas sensor, flexible heater, wire grid polarizer
[9]	Combined process (thermal shrinkage‐based F_mold_ weakening and mold stripping‐based F_mold_ weakening)	PMMA sacrificial layer, 150 °C for 25 sec by heat‐rolling system	NiO, Pt, WO_3_, Ag, Ge_2_Sb_2_Te_5_, Pd	wave, square, nut, zigzag, elliptical pattern	Colorless PI (CPI), slide glass, PET,	Memristive crossbar
[66]	Combined process (mechanical interlocking‐based F_substrate_ strengthening and thermoplasticity‐based F_substrate_ strengthening)	170 °C, 1min annealing for thermoplasticity‐based initial transfer, and 260 °C, 1hr for adhesion strengthening	Au, Ni, Pd, Ag, Cu	Line, asymmetric sidewall, suspended nanowire, dual‐layer line, dot, mesh	polyimide	Flexible and high‐temperature heater, asymmetric blind film
[99]	Combined process (plasma etching‐based F_mold_ weakening and covalent bonding‐based F_substrate_ strengthening)	SAM adhesive, 150 °C for 5 min on a hotplate, pressure by hand roller	Ag, Au, Al, TiO_2_, Pd, Pt, SnO_2_	Dot, cross, line, random rectangular pillar	Si wafer, SiO2 wafer, Al_2_O_3_ wafer, PMMA, PET, CPI, PDMS, Dragon Skin 10, thermoplastic polyurethane	Plasmonic color filter, hologram, EOT‐based strain sensor

## 2 Conventional Nanotransfer Printing

**Figure 2:**
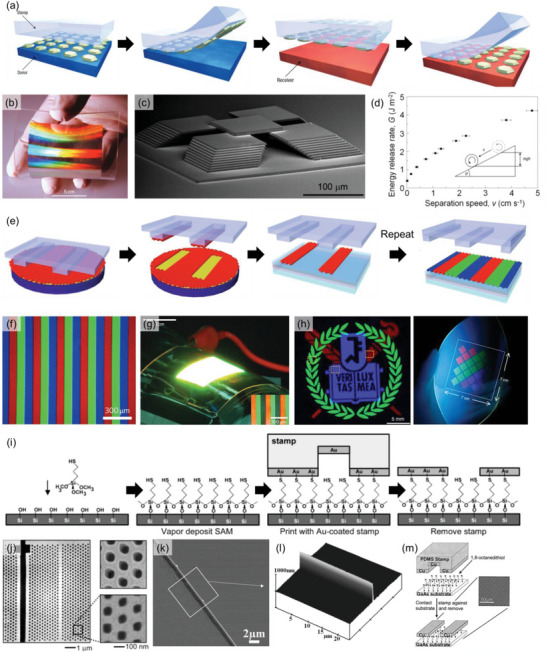
Conventional nTP processes. a) Schematic diagram showing kinetic control of adhesive force between the PDMS stamp and the target material. Reproduced with permission.^[^ ^12^ ^]^ Copyright 2006, Springer Nature Limited. b) Nanostructured metasurface transferred on the PDMS stamp. Reproduced with permission.^[^ ^10^ ^]^ Copyright 2011, Springer Nature Limited. c) 3D assembled nanostructures by sequential printing using the PDMS stamp. Reproduced under the terms of the CC‐BY‐4.0 license.^[^ ^11^ ^]^ Copyright 2010, The Authors, published by National Academy of Sciences. d) Peeling velocity‐dependent adhesion energy of the PDMS stamp. Reproduced with permission.^[^ ^12^ ^]^ Copyright 2006, Springer Nature Limited. e) Schematic diagram showing the inking and stamping process for patterning full‐color QD LED. f) Fluorescence micrograph of the printed QD patterns on the glass substrate. g) Photograph of the large‐area flexible QD LED fabricated by transfer printing. Reproduced with permission.^[^ ^5^ ^]^ Copyright 2011, Springer Nature Limited. h) High‐resolution RGB QD patterns fabricated on a flexible substrate by intaglio printing. Reproduced under the terms of the CC‐BY‐4.0 license.^[^ ^24^ ^]^ Copyright 2020, The Authors, Published by Springer Nature Limited. i) Schematic diagram showing self‐assembled monolayer (SAM) based nanotransferring method. Reproduced with permission.^[^ ^13^ ^]^ Copyright 2002, American Chemical Society. j) Example of the nanotransfer printing conducted by thiol‐based glue and gold nanopattern. Reproduced with permission.^[^ ^13^ ^]^ Copyright 2002, American Chemical Society. k,l) Example of the nanotransfer for sharp wall pattern conducted by SAM glue method, which requires high adhesion between substrate and target material. Reproduced with permission.^[^ ^14^ ^]^ Copyright 2008, WILEY‐VCH Verlag GmbH & Co. KGaA. m) Example of the different combinations of SAM glue: 1,8‐octanedithiol – Cu. Reproduced with permission.^[^ ^27^ ^]^ Copyright 2004, AIP Publishing.

Before the adhesive force at the interfaces (i.e., *F*_mold_ and *F*_substrate_) could be precisely controlled, researchers widely utilized flat polydimethylsiloxane (PDMS) as a donor substrate because of its low surface energy (≈19.8 mJ·m^−2^).^[^ ^20^ ^]^ When the equilibrium peeling occurs on a rigid disk, the peeling force *P* is given by:

(1) $$P^{2}=\frac{8\pi }{\left(1-\nu^{2}\right)}E\gamma a^{3}$$

where ν, *E*, γ, and *a* are the Poisson's ratio, Young's modulus, surface energy, and the radius of the disk. From the equation, it is clear that the low surface energy promotes facile delamination, making PDMS an attractive candidate as a donor substrate. However, if the adhesive interaction between PDMS and the target material is too strong or that between a receiver substrate and the target material is too weak, the transfer yield becomes significantly low. To alleviate this issue, Meitl et al. developed a way to modulate the adhesion and release of the target material by controlling the peeling rate of PDMS.^[^ ^12^ ^]^ Briefly, target materials are prepared on the donor substrate via conventional photolithography, solution casting, or micromachining. Then, the PDMS stamp is conformally contacted to the donor substrate and quickly peeled off. These “picked” target materials are then transferred to the receiver substrate when the PDMS stamp is laminated from the receiver substrate with a slow peeling rate (**Figure** **2**a). This rate‐dependent adhesion strength results from the viscoelastic response of the PDMS elastomer.^[^ ^11^, ^12^ ^]^ By utilizing the steel bar–PDMS rolling experiment, Meitl et al. proved that the separation energy increases with the increasing peeling rate (Figure 2d). As a result, the increased peeling rate causes the target material to adhere more strongly to the PDMS stamp than to the donor substrate.

This method significantly improved the transfer yield and pattern quality for practical applications that require high‐resolution patterns with excellent pattern periodicity with minimal defects. For example, Chanda et al. fabricated a flexible photonic nanomesh structure consisting of Ag/MgF_2_ alternating layers on the PDMS mold, which could be transferred to another substrate (Figure 2b).^[^ ^10^ ^]^ Since the transfer printing process is controlled by kinetically modulated adhesion force, it does not require high‐temperature processes, thus preventing any potential structural deformation that may arise from chemical or thermal degradation during conventional lithographic patterning. This is particularly important in photonics applications because light‐matter interaction typically requires long‐range‐ordered nanostructures with minimal defects. Moreover, sophisticated 3D nanostructures can be fabricated with sequential printing with the aforementioned PDMS stamping (Figure 2c).

Like microcontact printing, it is possible to transfer nanomaterials with high resolution (below 500 nm) using prepatterned stamps and inks. Instead of preparing a nanopatterned substrate, one can simply spin‐coat the target material on the donor substrate and harvest patterns using a nanopatterned PDMS stamp (Figure 2e). Then, this pattern is transferred to the receiver substrate via similar steps described in the previous paragraph. This process, called “inking and stamping” is beneficial when colloidal quantum dot (QD) patterning is required. For example, Kim et al. successfully fabricated a full‐color QD display by the sequential inking and stamping process of red, green, and blue QDs (Figure 2f).^[^ ^5^ ^]^ The fabricated device showed reasonably high luminance and current efficiency because the patterned QD layer showed similar film quality compared to the spin‐cast film. Moreover, these patterns can be transferred on curved, bumpy surfaces or even flexible substrates without significantly degrading device performance (Figure 2g). This method is still widely exploited for printing QDs to fabricate QD light‐emitting diodes.^[^ ^21^ ^]^ One of the issues of the inking and stamping process was the discrepancies between the original designs and the resulting pixel shapes due to the structural deformation of the elastomeric stamp.^[^ ^22^, ^23^ ^]^ Therefore, Choi et al. employed the intaglio transfer printing method for realizing polychromatic high‐resolution QD patterns up to 2460 pixels per inch on a flexible substrate (Figure 2h).^[^ ^24^ ^]^ Intaglio printing uses a flat PDMS stamp, but the negatively carved intaglio trench transfers the desired pattern to the stamp. Thus, large‐area full‐color QD patterns could be fabricated on a flexible substrate with a high pattern density, paving the way for integrating QD displays in wearable electronics.

In contrast with the above two methods that control the adhesion between mold and target material, the adhesion between substrate and target material can be controlled by adopting a self‐assembled monolayer (SAM) as a glue. Loo et al. reported the most famous combination of these materials: sulfur – Au,^[^ ^13^ ^]^ where the –OH group was formed on the surface of the substrate, such as Si wafer and PET film, and (3‐mercaptopropyl)trimethoxysilane (MPTMS) was coated onto the substrate based on the hydrolysis and condensation reaction^[^ ^25^ ^]^. MPTMS has an –SH group at the external end and this adheres to the Au pattern placed on the surface of the stamp (Figure 2i). As this method involves strengthening the adhesion between the receiver substrate and target material, it offers advantages in reducing surface diffusion and edge disorder, which are common “pain points” of conventional transfer methods such as microcontact printing.^[^ ^26^ ^]^ That is, the patterns with a higher resolution can be transferred by this method: it was confirmed that even sub‐100 nm patterns could be transferred through this method (Figure 2j). Due to the strong adhesion between the target material and substrate, this method can transfer patterns that necessitate high adhesion strength. For example, high‐aspect‐ratio patterns, which can be fabricated using the wall side of a deep‐trench mold, inevitably have a wide contact area with the mold; thus, the transfer of this pattern is relatively complex. In this case, the “SAM glue” method can provide sufficient adhesion for the transfer of the high‐aspect‐ratio pattern, and the sharp wall pattern was successfully transferred by this method (Figure 2k,l). Utilizing this method, named nanotransfer edge printing (nTEP), Xue et al. transferred the Au/SnO_2_ dual layer nanowall and applied it to create highly sensitive gas sensors.^[^ ^14^ ^]^ In addition to the most common attractive interaction (Thiol – Au bond), other combinations, such as 1,8‐octanedithiol (ODT) – Cu can also be employed (Figure 2m).^[^ ^27^ ^]^

In summary, there has been a great improvement in the transfer yield and pattern resolution of the nTP technique by the PDMS stamp‐based transfer printing method, leading to new possibilities for fabricating complex nanoarchitectures for diverse applications. These examples show the importance of having controllability on the adhesion energy for the practical use of nTP in real devices.

## 3 Recent Evolution of nTP Technology

Although the conventional nTP technique using elastomeric molds has been used to successfully fabricate nanoscale patterns through only a few simple processes, it has struggled with a critical trade‐off relationship between the resolution and pattern quality. These drawbacks mainly result from mechanical deformation of the elastomeric molds (e.g., buckling, roof collapse, and sagging by excessively applied mechanical pressure on a mold).^[^ ^28^ ^]^ The low elastic modulus of the elastomeric molds limits the pattern's ultimate resolution during replication.^[^ ^29^ ^]^ Moreover, the transfer yield becomes poor when the adhesive interactions between the mold and target material are relatively strong. For example, PDMS, a common material for an elastomeric mold, cannot be used for printing polar materials because of the hydrophobicity of its surface.^[^ ^30^ ^]^ These challenges necessitated the development of a new nTP principle that can replace the “inking and stamping” process, aiming to achieve uniform and reliable pattern transfers at high resolution.

Therefore, in the recent decade, researchers have explored interfacial adhesion force control methods between mold, pattern, and substrate to transfer more diverse patterns on various substrates with uniform and reliable processes. As explained in the introduction, when *F*_mold_ is weaker than *F*_substrate_, the nanopattern can be successfully transferred from the mold to the substrate. Depending on the concerning interfaces and corresponding strategies, they can be categorized into three groups as follows: i) *F*_mold_ weakening strategies (e.g., solvent‐injection‐assisted and mold stripping‐based adhesion weakening) mean weakening the adhesion force between mold and pattern to easily detach pattern from mold and ii) *F*_substrate_ strengthening strategies (e.g., adhesion strengthening based on adhesives, thermoplasticity, surface tension, and nanowelding) involve increasing the adhesive force between the pattern and substrate to firmly attach the pattern to the receiver substrate. In addition, iii) nTP techniques for combined processes of *F*_mold_ weakening and *F*_substrate_ strengthening have also been developed recently. In the subsequent chapter, we will explore the evolution of recent nTP research, including methods for fabricating nanopatterned molds, the distinctions between conventional nTP and more recently developed nTP approaches, as well as their respective advantages and disadvantages.

### 3.1 Fabrication Methods of Nanopatterned Mold

**Figure 3:**
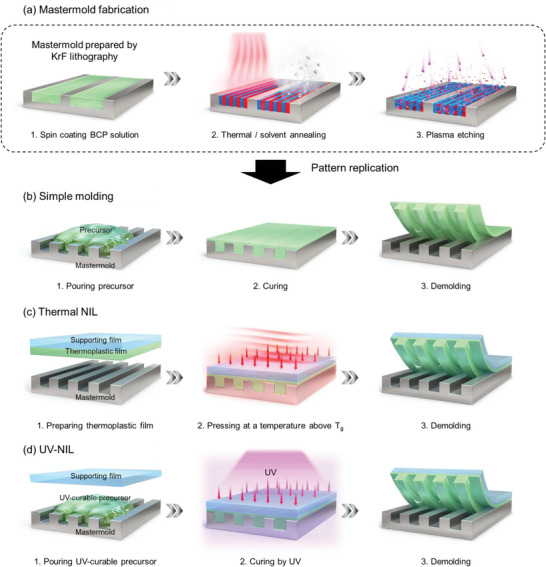
Preparation of the master mold and pattern replication. a) Schematic illustration showing the fabrication process of the Si master mold by KrF lithography and BCP self‐assembly. BCP self‐assembly is conducted by spin coating of BCP solution on the Si master mold, thermal or solvent‐vapor annealing of the BCP film, and selective block removal using plasma etching. b–d) Three representative fabrication methods of the nanopatterned mold using the Si master mold. Schematic illustration showing detailed fabrication processes based on i) simple molding: pouring precursor solution on the Si master mold, curing, and demolding, ii) thermal NIL: locating thermoplastic film on the Si master mold, pressing at a temperature above *T*_g_, and demolding, and iii) UV‐NIL: pouring UV‐curable precursor solution on the Si master mold, curing with UV light, and demolding,

The first step of nTP is the preparation of the nanopatterned master mold (generally, a Si wafer is used as a master mold). The most representative method to fabricate the master mold is krypton fluoride (KrF) lithography.^[^ ^31^, ^32^ ^]^ Although KrF lithography is of high cost, the master mold can be repeatedly reused for many cycles, ensuring cost‐effectiveness for the overall nTP implementation. As a complementary method to KrF lithography, directed self‐assembly of BCPs can be employed for the preparation of higher resolution master patterns with even sub‐20 nm pitch, as shown in **Figure** **3**a. BCP is a polymer where two (or more) polymer blocks are connected via covalent bonds.^[^ ^33^, ^34^, ^35^ ^]^ Mold fabrication using BCP begins with spin coating a BCP solution onto a pre‐patterned Si substrate, which serves as a guide template. The polymer chains in an as‐spun BCP film are typically disordered due to extremely short process time and limited chain mobility. Therefore, thermal annealing (at *T* > *T*_g_ of BCPs)^[^ ^36^, ^37^, ^38^ ^]^ or solvent exposure^[^ ^39^, ^40^, ^41^, ^42^ ^]^ to the BCP^[^ ^39^, ^40^, ^41^ ^]^ films is conducted to induce nanoscale phase separation and self‐assembly into well‐aligned patterns, which is directed by the Si guide pattern. Then, one of the blocks is selectively removed by plasma or wet etching to form a polymer pattern. The resulting pattern shows an extremely narrow width of ≈20 nm for polystyrene‐*b*‐poly(methylmethacrylate) (PS‐*b*‐PMMA), and even sub‐10 nm patterns have been demonstrated using polystyrene‐*b*‐polydimethylsiloxane (PS‐*b*‐PDMS) BCPs.^[^ ^43^, ^44^, ^45^, ^46^ ^]^ The ability to form an extremely high pattern density on a large substrate (> 4‐inch wafer^[^ ^47^ ^]^) with only a few simple processes (e.g., spin coating and annealing) makes BCP self‐assembly suitable for mold fabrication for the high‐resolution nTP. Despite the usefulness of BCP self‐assembly, there are remaining challenges that should be overcome for broader applications. For example, the number density of topological defects such as dislocation and bridging^[^ ^48^, ^49^ ^]^ is highly sensitive to the processing conditions and requires sophisticated optimization.^[^ ^50^ ^]^ Moreover, surface energy neutralization of the Si master mold using brush layer grafting is often necessary for a successful assembly of BCPs.^[^ ^51^ ^]^

The next step is replicating the master mold for fabricating the final nanopatterned mold, which will be used as a platform to deposit target materials. Given that the above‐mentioned limitations of the conventional nTP originated from the low Young's modulus of the elastomeric molds, researchers have adopted more diverse mold fabrication technologies that can be applied to more rigid but still flexible materials. Among various methods developed for decades, three representative fabrication processes, based on simple casting,^[^ ^15^, ^52^, ^53^, ^54^, ^55^ ^]^ thermal nanoimprint lithography (NIL),^[^ ^1^ ^]^ and UV‐NIL^[^ ^31^, ^32^, ^56^, ^57^, ^58^, ^59^, ^60^, ^61^, ^62^, ^63^, ^64^, ^65^, ^66^ ^]^ are widely used. In the following paragraph, depending on how the master mold is replicated into the polymer nanopatterned mold, these representative methods will be introduced with the different advantages and disadvantages.

First, replication from the Si master mold can be conducted by simple casting, which can be easily conducted without expensive equipment and advanced techniques, as shown in Figure 3a. In this method, a liquid precursor is poured into the master mold, cured, and detached from the master mold. Easily curable polymer precursors such as polyvinyl alcohol (PVA) in water,^[^ ^54^ ^]^ hyaluronic acid (HA) in water,^[^ ^15^ ^]^ and poly(methyl methacrylate) (PMMA) in acetone^[^ ^53^, ^55^ ^]^ and crosslinkable elastomer precursors^[^ ^52^ ^]^ are generally used, and the viscosity and polarity of the precursors are the key parameters for the success of replication. Too high viscosity or polarity mismatched with the master mold prevents the precursors from fully penetrating into the nanopatterns within the master mold. Although additional heat or pressure, including vacuuming, can be applied during the curing step to facilitate easy penetration and fast curing, there are still critical limitations in the slow process compared with the thermal NIL and UV‐NIL methods.

Second, the nanopatterned mold can be fabricated by thermal NIL using thermoplastics such as PVA^[^ ^1^, ^3^ ^]^ and PMMA,^[^ ^16^, ^65^ ^]^ as shown in Figure 3b. Above the glass transition temperature (*T*_g_) of thermoplastic, it behaves like a rubbery material and becomes more deformable. Therefore, by applying adequate pressure on the thermoplastic placed on the master mold at a temperature above *T*_g_, the nanopattern of the master mold can be engraved on the thermoplastic. It is beneficial because of the relatively fast process (< 10 min) and has the potential to be compatible with the conventional high‐throughput roll‐to‐toll process. However, some remaining issues are related to the required high pressure and defects originating from the secondary deformation during the demolding procedure or unevenly applied heat and pressure in large areas.

Third, UV‐NIL is another representative method of mold preparation. Although the overall process is similar to the thermal NIL, UV curable precursor, which is generally composed of polymer monomer (e.g., monomer of poly(urethane acrylate) (PUA)^[^ ^61^, ^67^ ^]^ or perfluoropolyether,^[^ ^3^ ^]^ and photoinitiators), is used, instead of the thermoplastic, to replicate the Si master mold, as shown in Figure 3c. Thus, it requires much lower pressure, has faster processes (< 3 min), and is free from defects during demolding compared to thermal NIL‐based mold fabrication. However, defects from air bubbles in precursor solution and UV transparency of the supporting film or the master mold should be considered during the process.

Here, currently used fabrication methods of the nanopatterned mold were discussed. These processes can be selectively applied in nTP, considering the characteristics of each method and compatibility with the target materials and the subsequent nTP process. The following chapters will introduce recent nTP based on the nanopatterned mold fabricated by these three representative methods.

### 3.2 *F*_mold_ Weakening

Controlling the adhesive force (*F*_mold_) between the mold and target material, in combination with advanced mold fabrication techniques introduced in the previous section, can alleviate the reliability and resolution limit of conventional nTP techniques by facilitating the delamination of the target material from the mold. One of the main advantages of reducing *F*_mold_ for the nTP process is that minimal (or zero) pressure is required during the process. Once the conformal contact is established between the target material and the substrate, the target material is spontaneously transferred to the substrate via the adhesion‐switching process. In addition, proper control of *F*_mold_ also enables pattern transfer to the arbitrary substrate, regardless of the hydrophobicity of the target material. As a result, high‐quality nanopatterns can be easily transferred to any desired substrate by facile delamination of the target material from the mold. This section will discuss the mechanisms and examples of nTP utilizing the *F*_mold_ weakening.

**Figure 4:**
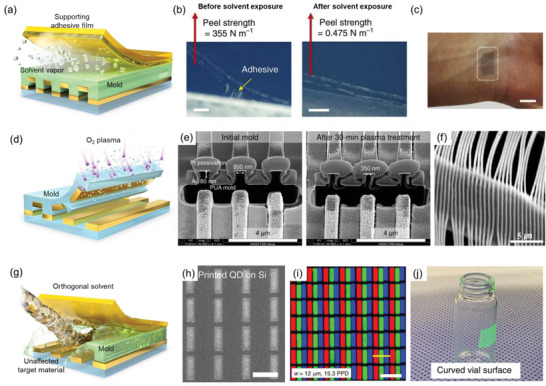
nTP based on *F*_mold_ weakening. a) Schematic illustration showing a lubrication process of solvent vapor molecules at the interface of the adhesive and the polymer mold. b) The peel strength test of the PMMA/PI tape before and after exposure to toluene vapor. c) Digital photograph of metallic nanowires transferred on a human wrist. Reproduced under the terms of the CC‐BY‐4.0 license.^[^ ^8^ ^]^ Copyright 2014, The Authors, published by Springer Nature Limited. d) Schematic illustration showing a mold stripping‐based adhesion weakening by plasma etching. e) Cross‐sectional SEM image showing a reduced contact area between Au and the PUA mold after plasma etching. Reproduced under the terms of the CC‐BY‐4.0 license.^[^ ^19^ ^]^ Copyright 2023, The Authors, published by Springer Nature Limited. f) Conformal Au nanowires transferred on textile fiber without crack or failure by water‐soluble HA mold, reproduced with permission.^[^ ^15^ ^]^ Copyright 2020, American Chemical Society. g) Schematic illustration showing a solvent‐immersion‐based transfer printing using an orthogonal solvent. h) Demonstration of full‐color QD patterning on a Si substrate. i) Digital photograph of a green QD film conformally transferred on a curved vial surface. Reproduced under the terms of the CC‐BY‐4.0 license.^[^ ^84^ ^]^ Copyright 2020, The Authors, Published by Springer Nature Limited.

Jeong et al. introduced the concept of interface‐targeted adhesion switching by exposing a bilayer (polystyrene (PS)/poly(4‐vinyl pyridine) (P4VP)) polymer thin film to the solvent vapor (**Figure** **4**a).^[^ ^8^ ^]^ The process called “solvent‐injection‐assisted nTP” (or S‐nTP) starts with replicating line patterns of the Si mold by spin coating the PS/P4VP polymer layer and detaching it using an adhesive film. Then, the target material is formed on the line‐patterned polymer layer by an obliquely angled deposition of metals by the e‐beam evaporation, which enables the fabrication of discrete nanostructures by utilizing the shadowing effect. For successful transfer of the target material from the adhesive to the substrate, one should weaken the *F*_mold_ between the adhesive and the polymer film. They utilized a phenomenon called “super‐lubrication” to reduce the friction at the interface, where the interpenetration of polymer chains is suppressed in the presence of a suitable solvent. Under this circumstance, an entropic penalty hinders polymer chains from being interpenetrated. Thus, swelling of polymers can significantly reduce the interfacial friction by a factor of ≈10^3^. Once the swollen PDMS gel pad supplying toluene vapor is attached to the polymer film, the solvent vapor molecules lubricate the PS/adhesive interface, and the adhesive film is naturally peeled off. The peel strength measurement demonstrates the role of solvent vapor before and after solvent vapor exposure. The peel strength between the PS film and the adhesive decreased from 355 to 0.475 N·m^−1^, showing nearly three orders of magnitude decrease of *F*_mold_ (Figure 4b). Later, they further developed the 2^nd^ generation S‐nTP using a polymethylmethacrylate (PMMA) single‐layer replica and acetone/heptane co‐solvent vapor, which makes the overall process much more straightforward and reliable.^[^ ^68^ ^]^ Instead of a swollen PDMS gel pad, the polymer replica/adhesive film was brought into a preheated solvent‐saturated chamber for 30 s for the *F*_mold_ weakening process. Given that the preparation of the swollen PDMS gel pad required more than 6 h,^[^ ^8^ ^]^ using the preheated solvent chamber significantly reduced the overall time for the printing process. The main advantage of *F*_mold_ weakening is that the transfer process does not require excessive heat or pressure.^[^ ^69^, ^70^, ^71^, ^72^ ^]^ This means that the *F*_mold_ weakening‐based nTP can deliver nanopatterns onto an arbitrary surface, including glass, fingernail, fruit skin, and human wrist (Figure 4c). The ability to transfer functional materials onto diverse substrates enables the fabrication of highly complex structures such as conformal metal nanowires on protruded semiconductor structures,^[^ ^73^ ^]^ plasmonic nanowires on soft contact lenses,^[^ ^68^ ^]^ and suspended single‐crystalline Si nanowires on rigid supports.^[^ ^74^ ^]^ In addition, 3D nanostructures can be fabricated by direct sequential printing of nanowires. For example, woodpile nanostructures can be prepared by printing second nanowire arrays by rotating the substrate with the first nanowire arrays by 90°, resulting in highly ordered nanostructures with a controlled gap size. These nanostructures can be utilized in several applications, including electronic devices, chemical sensors, and energy conversion devices, which will be discussed later.

Instead of reducing the friction between the adhesive and the polymer film, one can directly remove the polymer replica to make *F*_mold_ negligible (Figure 4d). This mold stripping‐based *F*_mold_ weakening can be done by selectively etching polymeric molds while leaving the inorganic target materials on the substrate.^[^ ^19^, ^75^, ^76^ ^]^ For example, Ahn et al. fabricated the 3D nanostructures by buckling Au nanowires on a pre‐stretched PDMS substrate.^[^ ^19^ ^]^ Here, they prepared Au nanowires on nanopatterned poly(urethane acrylate) (PUA) mold by e‐beam evaporation. Then, the PUA mold was selectively etched with oxygen plasma to reduce the contact area between the mold and Au. One can notice that the width of the PUA mold is significantly reduced from 800 to 350 nm after plasma etching without significant structural deformation of the target material (Figure 4e). The reduced contact area between the mold and the target material enables facile delamination of Au patterns to desired substrates. As a result, the successful transfer of Au nanowires on the PDMS mold to receiver substrates was achieved.

Another way for mold stripping‐based F_mold_ weakening can be done by dissolving molds with water using water‐soluble polymeric molds. Polyvinyl alcohol (PVA) is one of the most common materials used as a water‐soluble polymer replica. The high elastic modulus of PVA (1.9 GPa) compared to that of PDMS (1.8 MPa) ensures minimal pattern distortions and high pattern replication yield. Moreover, it is possible to rapidly form the PVA film using spin coating in less than 1 min, which is much faster than the time consumed for preparing the PDMS mold (≈30 min at 140 °C).^[^ ^77^ ^]^ For instance, a PVA template can replicate patterns on a 100‐mm‐diameter Si wafer, showing high uniformity and practicality of replicating patterns with PVA.^[^ ^78^ ^]^ Most importantly, its high solubility in water ensures facile and simple removal of polymer residue from the target material by simply washing the mold with water. In addition to PVA, one can also utilize hyaluronic acid (HA) as an alternative. The benefit of using HA originates from its high water‐solubility at room temperature (while PVA is typically dissolved in water at 70 °C) and its high viscosity to prevent the deformation of nanopatterns during the dissolution process. Ko et al. demonstrated the mold stripping‐based nTP technique by dissolving nanopatterned HA mold for fabricating nanostructured devices on the textile substrate (Figure 4f).^[^ ^15^ ^]^ Negligible mechanical pressure during the transfer process is beneficial for transferring highly brittle target materials or printing nanopatterns on flexible substrates. Thus, several morphologies (e.g., line, dot, mesh patterns) and materials (e.g., Pd, Ag, Au, Al, and SiO_2_) could be conformally printed on highly flexible fibers (e.g., nylon, cellulose, polyester, and spandex), leading to the applications to hydrogen gas sensors and nanostructured security patterns. Several examples have shown the versatility of the mold dissolution‐based nTP for transferring nanoscale features on a substrate.^[^ ^76^, ^79^, ^80^, ^81^, ^82^, ^83^ ^]^

Finally, to replace the conventional inking and stamping technique using elastomeric molds, Nam et al. reported an immersion transfer‐printing (iTP) technique featuring solution‐based deposition, patterning, and printing of target materials (Figure 4g).^[^ ^84^ ^]^ Although the aforementioned mold stripping techniques can fabricate aligned metal nanopatterns prepared by the vacuum deposition process, it has been challenging to form ordered nanostructures from colloidal nanocrystals that are highly vulnerable to physical and chemical damages from heat, solvent, and plasma. Especially, unlike metal nanostructures that are robust to most solvents (e.g., water, ethanol, and acetone), colloidal nanocrystals might be dissolved by the solvents due to the presence of capping agents. Nam et al. developed a process called “one‐step programmed self‐assembly” for patterning quantum dots (QDs) to simultaneously deal with the stability and picking issues of patterning QDs. First, the surface of the nanopatterned Si mold was modified by the hydroxyl‐group‐functionalized PDMS brush to facilitate facile delamination of QDs from the mold. Since QDs are typically passivated with non‐polar capping agents such as oleic acid, the QD solution is also dispersed in a non‐polar solvent such as heptane. Therefore, direct spin coating of the heptane‐based QD solution on a PDMS‐treated Si mold would form a flat film without any patterns due to the high wettability of heptane on the PDMS surface. To promote a spontaneous pattern formation of QDs during spin coating, they utilized a binary solvent consisting of heptane and toluene, where toluene acts as a dewetting‐inducing solvent. After adding toluene, the spinodal dewetting of the QD solution on the PDMS‐treated Si mold prevents film formation on the mesa region, while the geometrical confinement effect leads to the precise positioning of QDs in trenches. The PMMA transfer media picks the patterned QDs, and the whole QD/PMMA layer is peeled off from the Si mold with adhesive tape. The transfer of the QDs to the substrate is performed by mold dissolution‐based adhesion switching, which is a process of immersing QD/PMMA/adhesive tape in an orthogonal solvent, acetone. Because of the polar characteristics of acetone molecules, PMMA chains are dissolved in acetone, and the adhesion between PMMA and adhesive becomes significantly weaker when immersed in acetone. The interaction between non‐polar capping agents and polar solvents is negligible; therefore, QDs capped with oleic acid have very low adhesive energy with acetone. Thus, the physical adhesion between QDs and the substrate becomes strong enough to switch the adhesion from PMMA to the substrate. The iTP technique allows for the fabrication of an ultrahigh‐resolution full‐color QD array with high luminance, owing to the minimal damage to QDs during the transfer process (Figure 4h,i). Moreover, the QD patterns can be transferred to diverse substrates, including curved vial surfaces, flexible plastic substrates, leather, and animal skins (Figure 4j). As a result, the low printing fidelity and reliability issues of conventional PDMS‐based QD stamping could be overcome for developing highly efficient QLED displays with an ultrahigh pattern density beyond eye‐limiting resolution. Furthermore, iTP demonstrated the ultimate resolution of QD patterning – “single‐QD width” patterns – by utilizing the PS‐b‐PDMS BCP self‐assembly master mold described earlier.

Overall, *F*_mold_ weakening, including solvent‐injection‐assisted lubrication, mold‐dissolution, and orthogonal‐solvent‐assisted adhesion switching, can be widely applied to transfer high‐resolution patterns with excellent periodicity and uniformity.

### 3.3 *F*_substrate_ Strengthening

Establishing effective adhesion between the target material and the receiving substrate is a critical factor for the successful transfer and utilization of the target material. However, the methods mentioned above, such as weakening the *F*_mold_, cannot strengthen the adhesion of the target material to the receiving substrate. To handle this issue, the nTP method based on *F*_substrate_ strengthening has also been actively researched. Previously, the idea of *F*_substrate_ strengthening was utilized in thiol‐based Au transfer methods.^[^ ^13^, ^14^ ^]^ However, they are highly restricted to specific pairs of materials. Nowadays, more general methods to achieve the reinforcement of *F*_substrate_ have been widely introduced. This section introduces four methods that have been utilized for *F*_substrate_ strengthening: adhesive, thermoplasticity, surface tension, and nanowelding.

The first method is the adhesive‐based *F*_substrate_ strengthening. As mentioned above, *F*_substrate_ strengthening was achieved by specific pairs of attractive interactions, such as thiol and Au. Instead, adhesives have been widely researched and utilized. In the simplest method, similar to the conventional tape based on the pressure‐sensitive adhesive, the contact face of the substrate has been fabricated with an adhesive‐like feature and utilized in nTP.^[^ ^57^, ^75^, ^85^ ^]^ For example, Wang et al. utilized a tape as the substrate for the nTP process.^[^ ^85^ ^]^ They directly transferred the target material to the tape (substrate) and used it as a flexible and wearable nanophotonic device. In addition, materials that have been widely adopted as adhesives, such as NOA61,^[^ ^1^, ^86^ ^]^ PMMA,^[^ ^76^, ^87^ ^]^ NIL‐6000E,^[^ ^62^ ^]^ and epoxy.^[^ ^88^ ^]^ also have been coated onto the substrate and adopted as the adhesive between the target material and substrate. Among them, Li et al. picked the NOA61 as an adhesive, selectively transferred the electrodeposited Au nanopattern onto the substrate, and utilized it for IgG‐based plasmonic biomolecule detection.^[^ ^1^ ^]^

**Figure 5:**
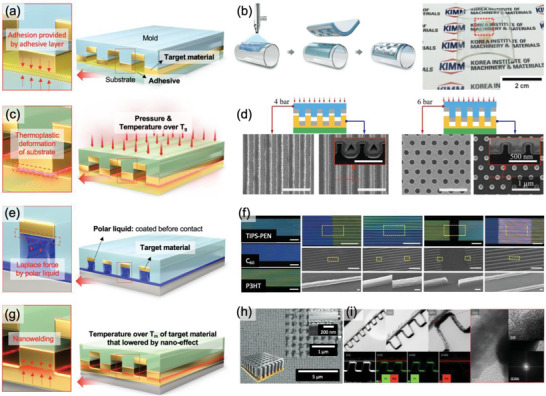
nTP based on *F*_substrate_ strengthening. a) Schematic illustration showing an adhesive‐based *F*_substrate_ strengthening. b) The advantage of the adhesive‐based *F*_substrate_ strengthening: processible onto curvy surface. Reproduced with permission.^[^ ^18^ ^]^ Copyright 2017, Royal Society of Chemistry. c) Schematic illustration showing a thermoplasticity‐based *F*_substrate_ strengthening. d) The advantage of the thermoplasticity‐based *F*_substrate_ strengthening: controllable depth of the transferred target material into the substrate. Reproduced with permission.^[^ ^32^ ^]^ Copyright 2020, American Chemical Society. e) Schematic illustration showing a Laplace force‐based *F*_substrate_ strengthening. f) The advantage of the Laplace force‐based *F*_substrate_ strengthening: organic nanowire‐compatible process with multiple forms of heterogeneous junctions. Reproduced with permission.^[^ ^92^ ^]^ Copyright 2016, WILEY‐VCH Verlag GmbH & Co. KGaA. g) Schematic illustration showing a nanowelding‐based *F*_substrate_ strengthening. h) The advantage of the nanowelding‐based *F*_substrate_ strengthening: multi‐layer transferring. Reproduced with permission.^[^ ^61^ ^]^ Copyright 2017, WILEY‐VCH Verlag GmbH & Co. KGaA. i) The specialty of the nanowelding‐based *F*_substrate_ strengthening: low electrical resistance between transferred layers. Reproduced with permission.^[^ ^17^ ^]^ Copyright 2020, American Chemical Society.

However, making thin adhesive layers with these materials is challenging, which inevitably affects the overall physical properties of the substrate. In other words, they should be accompanied by changes in the original physical property of the substrate, which may sometimes cause critical issues. For example, to be utilized in optical applications, the optical properties of the substrate should be analyzed. However, the use of a thick adhesive may alter the optical properties of the film, resulting in different optical characteristics from the intended design. To avoid this, *N*‐[3‐(Trimethoxysilyl)propyl]ethylenediamine (mr‐APS1), can be a feasible alternative, which has been widely used in the research of Hwang et al.^[^ ^31^, ^59^ ^]^ The working mechanism of the mr‐APS1 is different from those of the abovementioned adhesives. It constructs a SAM layer on the surface of the substrate and forms a covalent bond with the target material (**Figure** **5**a). As the adhesive thickness required for mr‐APS1 is extremely thin, consisting of only a single layer of molecules, it does not significantly impact the substrate's physical properties. In addition, this adhesive is based on the SAM layer and thus can be efficiently utilized for curved surfaces (Figure 5b). Initially, mr‐APS1 adhesion was adopted for SiO_2_ and substrate bonding at its early stage of usage.^[^ ^59^ ^]^ However, it has since been expanded to enable adhesion between the substrate and native oxide of various metal surfaces,^[^ ^31^ ^]^ thus ensuring material versatility. However, to form covalent bonds, a high temperature of 150 °C is required, and it may be restrictive for some polymer substrates with low thermal stability.

The second method is thermoplasticity‐based *F*_susbstrate_ strengthening. It is well known that the thermoplastic polymer shows liquidity with an increase in temperature over *T*_g_. When sufficient pressure is applied over the target material that contacts the substrate, at a temperature over *T*_g_, the surface of the substrate and target material uniformly contact and induce a higher *F*_substrate_. In addition, the temperature difference during and after transfer induces mechanical interlocking.^[^ ^16^ ^]^ These factors have been known as the driving force of the *F*_substrate_ strengthening by thermoplasticity of the substrate (Figure 5c). This method is easily applicable to various thermoplastic polymer substrates such as poly(methyl methacrylate) (PMMA),^[^ ^16^, ^32^, ^65^, ^82^, ^83^ ^]^ polyvinyl alcohol (PVA),^[^ ^3^, ^54^ ^]^ and polyamic acid (PAA).^[^ ^66^ ^]^ Furthermore, the fluidity of the substrate can be utilized to control the depth of the transferred target material onto the substrate. Zhao et al. confirmed that the transfer pressure, temperature, and duration affected the target material's final depth (Figure 5d).^[^ ^16^ ^]^ This method can be utilized for facile nTP onto a thermoplastic substrate without additional adhesives.

The third method is surface tension‐based *F*_substrate_ strengthening. Most of the abovementioned methods require UV exposure (e.g., NOA61) or high temperature (e.g., PMMA), making them difficult to utilize for the transfer of organic materials. To address this issue, Hwang et al. proposed liquid‐bridge‐mediated nanotransfer molding (LB‐nTM).^[^ ^89^ ^]^ This method involves the following steps: first, organic ink is applied onto a nanopatterned mold with low surface energy. The ink is selectively dried onto the grooved side of the mold to form organic nanowires. Next, a polar liquid is coated onto a substrate with high surface energy, and the mold containing the solidified organic nanowires is brought into contact. The organic nanowire and polar liquid come into contact, and the Laplace force induced by the polar liquid results in adhesion between the organic nanowire and substrate^[^ ^89^, ^90^, ^91^, ^92^ ^]^ (Figure 5e). The same group proposed the inkjet printing‐assisted LB‐nTM method as an advancement to this technique.^[^ ^92^ ^]^ The inkjet printing method enables the dispensing of the liquid with various organic materials on demand and is utilized to fabricate various pairs of organic nanowires (Figure 5f). Various morphologies were successfully fabricated, such as the mixture of two organic materials, gap‐formed and cross‐contacted organic nanowires. This method offers an advantageous approach for forming and transferring organic nanowires onto arbitrary substrates.

The fourth method is the nanowelding‐based *F*_substrate_ strengthening. In the case of the sub‐30 nm metal films, the melting point can be lowered due to the nano‐effect. Through this melting‐point depression, a stable junction between different metal layers can be formed. Zhao et al. utilized this phenomenon to transfer metal nanopatterns onto arbitrary metal surfaces (Figure 5g).^[^ ^2^, ^17^, ^61^, ^63^, ^67^ ^]^ After transferring the first metal layer onto the substrate through the abovementioned methods (adhesive and substrate thermoplasticity), additional metal nanopatterns can be transferred by nanowelding.^[^ ^61^ ^]^ Especially, this method has advantages for multi‐layer formation since the pre‐transferred metal can be adopted as the adhesive for the newly transferred metal pattern. Therefore, the *F*_substrate_ remains constant and concrete with continuous additional transfer, enabling the stable multi‐layer formation of metal nanopatterns (Figure 5h). Furthermore, the contact between target materials (metal nanopatterns) is more electrically stable than other methods since they form robust metal bonds; this transfer shows the low electrical contact resistance between layers (Figure 5i).^[^ ^17^ ^]^ Therefore, this method can be widely adopted for electrical applications such as electrical heaters and resistance‐based sensors.

In conclusion, with the *F*_mold_ weakening, although the applicable range of the nanotransfer printing can be widened, external disturbance can still easily occur. Through *F*_substrate_ strengthening, the stability of the nanotransfer printing can be dramatically increased. In addition, the stability of the final device can also be improved through *F*_substrate_ strengthening. Therefore, the *F*_substrate_ strengthening method can be advantageous for applications under harsh environments or final devices that require high reliability.

### 3.4 Combined Process

Despite significant progress in research on nTP based on the individual control of interfacial adhesion force (e.g., *F*_mold_ weakening and *F*_substrate_ strengthening), there are remaining fundamental bottlenecks ascribed from the nTP working mechanism. For example, for in‐mold stripping‐based nTP technology, the surface tension of the liquid etchants possibly causes detrimental effects on the nTP yield (e.g., aggregation or delamination of the transferred nanopattern) because the *F*_substrate_ is predominantly comprised of van der Waals forces, which are relatively weak.^[^ ^93^ ^]^ Furthermore, adhesive‐based nTP technology generally has a relatively weak *F*_substrate_ compared to thermoplasticity‐based nTP. This makes their use in nTP difficult when intrinsic *F*_mold_ is high. Therefore, to overcome these limitations, recent research is focused on developing more advanced nTP methods by adopting multiple strategies simultaneously, such as the combined process of mold stripping‐based nTP and adhesive‐based nTP.^[^ ^19^ ^]^ These combined processes allow one to synergistically utilize the advantages of each method, enabling more versatile and universal nTP,^[^ ^69^, ^80^, ^94^, ^95^, ^96^ ^]^ Overall, studies are focusing on improving the morphological/material diversity of the target materials and substrates with reliable large‐area processes, and they can be generally classified into three different groups: multi‐step processes of i) the repeated *F*_mold_ weakening^[^ ^9^ ^]^ or ii) the repeated *F*_substrate_ strengthening,^[^ ^66^ ^]^ and iii) combination of *F*_mold_ weakening and *F*_substrate_ strengthening.^[^ ^19^ ^]^ In the following paragraphs, representative examples are presented to show how the abovementioned limitations can be overcome and to discuss recent synergistic strategies for introducing the combined process.

**Figure 6:**
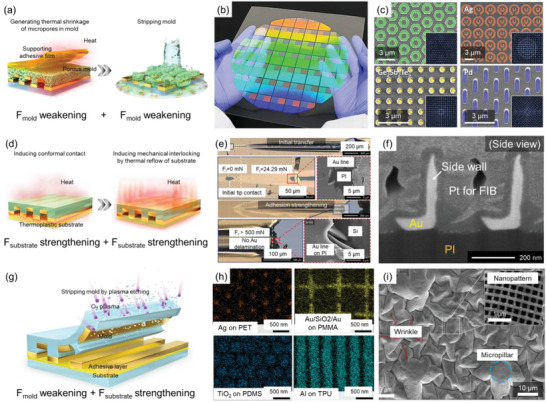
nTP based on the combined processes of *F*_mold_ weakening and *F*_substrate_ strengthening. a) Schematic illustration of the representative fabrication mechanism of nTP based on repeated *F*_mold_ weakening (thermal shrinkage‐based *F*_mold_ weakening and mold stripping‐based *F*_mold_ weakening). b) Results of nTP on the 8‐inch wafer showing the reliable large‐area process. Reproduced under the terms of the CC‐BY‐4.0 license.^[^ ^9^ ^]^ Copyright 2020, The Authors, published by AAAS. c) Results of nTP with various complex pattern geometries with diverse materials for demonstrating morphological/material diversity of the target materials. Reproduced under the terms of the CC‐BY‐4.0 license.^[^ ^9^ ^]^ Copyright 2020, The Authors, published by AAAS. d) Schematic illustration of the representative fabrication mechanism of nTP based on repeated *F*_substrate_ strengthening (thermoplasticity‐based *F*_substrate_ strengthening and imidization‐based *F*_substrate_ strengthening). e) Results of scratch test for demonstrating strong adhesion of nanopatterns with substrates compared with the nanopatterns produced by nTP without imidization. Reproduced under the terms of the CC‐BY‐4.0 license.^[^ ^66^ ^]^ Copyright 2021 The Authors, published by Elsevier B.V. f) Results of nTP with asymmetric sidewalls showing the morphological diversity of the target materials. Reproduced under the terms of the CC‐BY‐4.0 license.^[^ ^66^ ^]^ Copyright 2021 The Authors, published by Elsevier B.V. g) Schematic illustration of the representative fabrication mechanism of nTP based on the combination of *F*_mold_ weakening and *F*_substrate_ strengthening (mold stripping‐based *F*_mold_ weakening and adhesive‐based *F*_substrate_ strengthening). h) Results of nTP with four optical materials on diverse substrates. Reproduced with permission.^[^ ^97^ ^]^ Copyright 2023, American Chemical Society. i) SEM image of micro/nano hierarchical structures fabricated by combined processes. Reproduced with permission.^[^ ^3^ ^]^ Copyright 2021, Elsevier Ltd.

First, two different *F*_mold_ weakening strategies can be applied step‐by‐step to develop a reliable large‐area nTP technology with high morphological/material diversity of the target materials and substrates. Park et al. reported a thermally assisted nTP process that can easily produce well‐ordered nanostructures on an 8‐inch wafer using a heat‐rolling press system^[^ ^9^ ^]^ The study focuses on two types of adhesion forces related to the mold, which are defined as *F*_mold,1_ and *F*_mold,2_, as shown in **Figure** **6**a. *F*_mold,1_ pertains to the adhesion force between the mold and adhesive layer, and *F*_mold,2_ refers to the adhesion force between the mold and target material, as shown in Figure 6a. Thermal shrinkage‐based *F*_mold,1_ weakening (thermal shrinkage of micropores in porous PMMA mold) was first introduced to gently remove the adhesive film, while the adhesive film was used to handle the nanopatterned mold, enable conformal surface contact, and apply uniform heat/pressure. Then, mold stripping‐based *F*_mold,2_ weakening was sequentially applied to remove the remaining mold, as shown in Figure 6a. The authors claimed that the use of two distinct *F*_mold_ weakening strategies allows for the production of diverse complex pattern geometries (e.g., wave, square, nut, zigzag, and elliptical nanostructures) with a range of materials (e.g., WO_3_, Ag, Ge_2_Sb_2_Te_5_, and Pd). In addition, they claim that the method ensures a reliable large‐area nTP process, as shown in Figure 6b,c.

Second, two different *F*_substrate_ strengthening strategies also can be used to dramatically increase *F*_substrate,_ which would be particularly beneficial in improving the morphological diversity and mechanical robustness. Jeong et al. reported robust nanotransfer printing based on imidization‐induced mechanical interlocking,^[^ ^66^ ^]^ as depicted in Figure 6d. They claimed that thermoplasticity‐based *F*_substrate_ strengthening in a polyamic acid substrate facilitated conformal contact, which maximized the van der Waals force. In addition, further mechanical interlocking could be generated by the thermal reflow of the polymer during the imidization process, leading to a more robust *F*_substrate_ than before the imidization process, as demonstrated in the scratch test shown in Figure 6e. Owing to the exceptional robustness of the method, various 3D nanostructures with small contact areas with the substrate, such as asymmetric sidewalls, can be successfully transferred, as depicted in Figure 6f.

Third, combining *F*_mold_ weakening and *F*_substrate_ strengthening can simultaneously achieve a universal nTP^[^ ^97^ ^]^ or requirements in specific applications.^[^ ^3^ ^]^ For example, Ahn et al. recently reported a universal and reliable nTP technique that can be used for four optical materials (i.e., Ag, Au/SiO_2_/Au, Al, and TiO_2_) and nine different substrates, including rigid, flexible, and stretchable substrates, as shown in Figure 6g,h.^[^ ^97^ ^]^ In this study, a universal nTP technique was realized by plasma etching‐based *F*_mold_ weakening and covalent bonding‐based F_substrate_ strengthening, enabling the reliable modulation of the relative adhesion forces. In another study, Ahn et al. reported a two‐step nTP method that allows for the fabrication of morphology‐controllable wrinkled micro/nano hierarchical structures.^[^ ^3^ ^]^ Here, mold stripping‐based nTP was used to transfer various nanopatterns on a micropatterned substrate (i.e., to improve morphological diversity of the substrate), and adhesive‐based F_substrate_ strengthening was adopted to secure original nanopatterns when the mold is dissolved in the liquid etchant. This process was used to develop a pressure sensor.

Overall, researchers are actively striving to achieve both material and morphological versatility with a reliable large‐area process by utilizing the synergistic effects of combined adhesion control techniques. The ultimate goal is to harness the synergistic effects of these techniques and enable the effective use of nTP technologies across a wide range of fields, particularly in the development of nanophotonic, physical, and chemical devices in the near future. Further research in this area is crucial to facilitate the widespread adoption of nTP technologies and their successful integration into various applications.

## 4 Emerging Applications

With the expanding diversity of applicable target materials and substrates nTP, a growing number of fields have embraced nTP technology for its various advantages. In addition to its large area, high throughput, high uniformity, and high spatial resolution fabrication process, the unique physicochemical characteristics of the nanostructures produced by nTP have garnered significant attention in niche applications.

First, the well‐ordered nanostructures produced by nTP can induce plasmonic effects, making them useful in a range of applications such as plasmonic cut‐off filters (e.g., color filters), surface‐enhanced Raman spectroscopy (SERS), holograms, polarizers, and plasmonic biosensors. Second, thin metal nanopatterns fabricated by nTP exhibit high electrical conductance with high mechanical flexibility, making them useful in strain sensors and triboelectric nanogenerators (TENGs). Third, Uniform nanomesh structures produced by nTP have high electrical conductance with high optical transparency, which is beneficial in applications such as transparent heaters and electrodes. Finally, well‐arrayed nanostructures produced by nTP have high surface area‐to‐volume ratios, making them useful in gas sensors and catalysts.

In this section, these devices are classified into three groups based on their fundamental purposes: physical devices, nanophotonic devices, and chemical devices. The versatility and potential of nTP in various applications make it a promising technology with significant opportunities for further advancements.

### 4.1 Physical Devices

A highly ordered nanoscale pattern can yield various physical properties that have been favorable to high‐performance physical devices. For instance, one of the most promising features enabled by nTP is flexibility, wherein downsized nanopatterns with sub‐100 nm scale thickness can be transferred onto a diverse range of flexible substrates. As a result, the overall transferred output typically follows the flexibility of the substrate. Physical devices can be roughly divided into two sub‐categories: electrical devices and optical devices. While nTP has shown promise in the manipulation of nanoscale patterns for the development of nanophotonic effect‐based optical devices, this will be discussed in a separate section. This section will focus on the application of nTP in the development of flexible energy harvesters, heaters for electrical devices, and optical instruments for ray optics.

**Figure 7:**
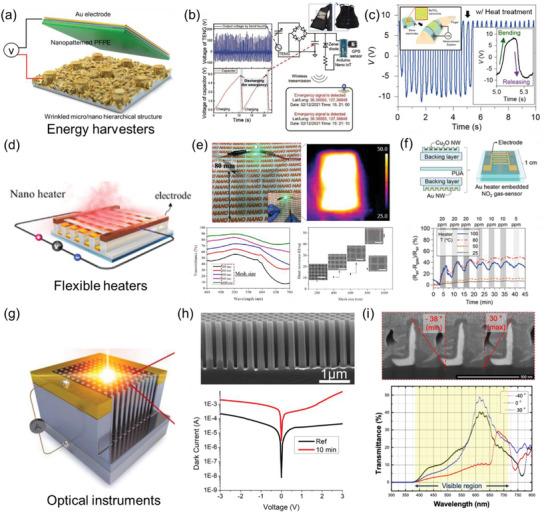
Application of nTP as physical devices. a) Schematic illustration of the TENG fabricated based on micro‐pattern molding and nTP method. Reproduced with permission.^[^ ^3^ ^]^ Copyright 2021, Elsevier Ltd. b) Utilization of the TENG fabricated by nTP method to the ocean environment energy harvesting. Reproduced with permission.^[^ ^65^ ^]^ Copyright 2022, WILEY‐VCH Verlag GmbH & Co. KGaA, Weinheim. c) Bio‐energy harvesting by piezoelectric generator. Reproduced with permission.^[^ ^96^ ^]^ Copyright 2017, American Chemical Society. d) Schematic illustration of the nTP‐based heater. Reproduced with permission.^[^ ^17^ ^]^ Copyright 2020, American Chemical Society. e) Control of optical transmittance and electrical conductance based on the morphology of the nanotransferred patterns, and their application as a flexible and transparent heater. Reproduced with permission.^[^ ^16^ ^]^ Copyright 2021, American Chemical Society. f) Gas sensor with temperature modulation operated based on an nTP‐based flexible heater. Reproduced with permission.^[^ ^94^ ^]^ Copyright 2018, American Chemical Society. g,h) Schematic illustration of the vertically aligned Si nanowire fabricated by nTP and following metal‐assisted chemical etching, and its usage as a photodetector. Reproduced with permission.^[^ ^64^ ^]^ Copyright 2022, American Chemical Society. i) L‐shaped nanowall structure utilized as incident angle‐selective optical blind. Reproduced under the terms of the CC‐BY‐4.0 license.^[^ ^66^ ^]^ Copyright 2021 The Authors, published by Elsevier B.V.

The first application of nTP to be discussed is in the development of flexible energy harvesters. Amongst various features of the nTP, a high SA:V ratio can be advantageously applied to flexible energy harvesters. For example, a TENG, which generates electricity based on the contact between different materials, needs a wider contact area for higher energy harvesting performance. The high SA:V that can be achieved by the nTP can substantially enhance the performance of the TENG. As an example of this usage, Ahn et al. fabricated the TENG based on a hierarchical micro‐nano structure. In this example, the micropattern was fabricated by micro‐molding, and the nano‐pattern was transferred onto it by adhesive‐based *F*_substrate_ strengthening. This fabrication method can increase the contactable surface area of the functional film and thus dramatically enhance the performance of the TENG (**Figure** **7**a).^[^ ^3^ ^]^ Furthermore, the functional film with a nanotransferred pattern comprises not only frequently adopted metals such as copper, silver, and nickel, but also rare metals such as palladium (Pd). The high chemical stability of Pd enables the energy harvesting device be applied in the ocean environment.^[^ ^65^ ^]^ Since Pd is not corroded by seawater, it can be applied for TENG in ocean environments, such as energy‐harvestable life jackets (Figure 7b). The harvested energy by the proposed TENG was used for SOS signal generators. Seo et al. also proposed bioenergy harvesting applications fabricated by combined methods of *F*_mold_ weakening and *F*_substrate_ strengthening.^[^ ^96^ ^]^ The piezoelectric property of the BaTiO_3_ was used to harvest the bio‐mechanical energy at the finger joint. To achieve this, a two‐step sacrificial‐layer etching technique (T‐SET) based nTP method was proposed. This approach combines both *F*_mold_ weakening and *F*_substrate_ strengthening to transfer a BaTiO_3_ nano line onto a flexible substrate. One of the key benefits of this method is that it allows for annealing of the nanopattern before the transfer process, resulting in an improvement in the piezoelectric property of the BaTiO_3_ nanopattern. (Figure 7c).

The second application of the nTP process is the flexible heater. Conventional flexible heaters, owing to their easy fabrication and high conductivity, have predominantly utilized the silver nanowire (AgNW) coating method. However, these flexible heaters often suffer from the problem of pattern non‐uniformity, which can result in an uneven heating profile. In contrast, the use of nTP in the fabrication of heaters can overcome this issue, enabling uniform pattern transfer across a wide area while still retaining the benefits of AgNW‐based flexible heaters (Figure 7d).^[^ ^17^ ^]^ Additionally, depending on the pattern, the physical properties of the film, such as transparency and electrical conductance, can be modulated. The transparency can be controlled by the fill ratio,^[^ ^16^ ^]^ and the electrical conductance can be controlled by the fill ratio and the thickness of the pattern.^[^ ^66^ ^]^ This can be confirmed by the research of Zhao et al.^[^ ^16^ ^]^ They fabricated a nanomesh pattern by thermoplasticity‐based F_substrate_ strengthening. This paper confirmed that the morphology of the mesh pattern could uniformly modulate the transparency and electrical conductance with a negative correlation (Figure 7e). With a morphology of pitch = 1600 nm and width = 100 nm, a transmittance of about 90% could be achieved. At this point, the sheet resistance was measured to be 48 Ω sq^−1^. In addition to imparting specific features to the heater, the incorporation of different elements is one of the most promising applications of flexible heaters. As an example of the flexible heater‐based system, Seo et al. proposed the flexible heater‐based gas sensor (Figure 7f).^[^ ^94^ ^]^ This sensor was fabricated with combined methods of *F*_mold_ weakening (mold stripping‐based) and *F*_substrate_ strengthening (adhesive‐based). Most of the metal oxide‐based gas sensors show good sensing performance under high temperatures, and this environment was given by including flexible heaters in the proposed research. The enhancement of gas sensing performance depending on the temperature increase was confirmed in this research.

The third application of the nTP process is the optical instruments for ray optics. As mentioned above, nano‐effect‐based optical features such as extraordinary optical transmission (EOT) will be explained in the next section; hence, this part will simply explain the applications concerning light path modulation. Here, two examples will be explained: vertically aligned nanowire structure to increase the SA:V and L‐shaped nanowall structure for incident‐angle selective transmission characteristics. Zhao et al. suggested the first example: an increase of the SA:V by vertically aligned nanowire structure.^[^ ^64^ ^]^ The Au pattern was directly transferred onto the Si wafer to fabricate this structure, and the Si wafer's metal‐assisted chemical etching (MACetch) was conducted. Through this method, the highly aligned vertical Si nanowire forest could be fabricated and applied as a photodetector (Figure 7g). Since the Si is inherently applied as the photodetector, producing a photocurrent based on the external light input, the increase in SA:V with the external light can enhance its performance as a photodetector (Figure 7h). Jeong et al. proposed the second example: an L‐shaped nanowall structure for incident‐angle selective transmission characteristics.^[^ ^66^ ^]^

It was fabricated based on the side deposition of the nanoline pattern, and thus the transfer of this structure needs relatively high adhesion. Thus, in this research, the combined methods of two *F*_substrate_ strengthening methods were adopted (thermoplasticity‐based + imidization‐based). The L‐shaped nanowall structure exhibits varying fill ratios based on the angle of incident light, resulting in different transmittance levels depending on the angle of incident light. This unique property was leveraged to develop an incident angle‐selective optical blind (as depicted in Figure 7i), which demonstrated a significant 35% difference in transmittance levels for different incident angles of light.

In summary, nTP has significant potential to enhance the performance of a diverse range of physical devices, including both electrical and optical devices. While there have been numerous examples of nTP applications in this field, this section has highlighted three representative applications: flexible energy harvesters, flexible heaters, and light path modulating devices. Moreover, the review exclusively concentrated on the transfer of nano‐sized components, omitting the discussion of the fabrication of micro‐LED arrays, which stands as a highly promising utilization of the transfer technique.^[^ ^98^ ^]^ Nevertheless, it is worth noting that the nTP methods introduced are not confined solely to the nano‐scale realm; they can also be effectively employed at the micro‐scale. Hence, these methods hold considerable potential for the production of micro‐LEDs. Furthermore, as micro‐LEDs continue to shrink in size towards the nano‐scale, these methods are poised to remain relevant and valuable in future applications.

### 4.2 Nanophotonic Devices

Nanophotonic devices are considered to be one of the most promising applications of nTP. This unique class of physical devices offers a wide range of fascinating features as optical components.^[^ ^88^, ^99^ ^]^ Therefore, we explore the nanophotonic devices in this section as a separate chapter. Nanophotonic devices refer to devices that can manipulate light on a nanoscale, utilizing the properties of light‐matter interaction at the nanoscale. Most nanophotonic devices are generally based on the plasmonic effect, which occurs when light interacts with a metal nanostructure. This interaction creates surface plasmon polaritons (SPPs), which are collective oscillations of electrons at the metal‐dielectric interface. These SPPs can enhance the interaction between light and small structures, leading to unique optical properties. In the realm of nanophotonics, significant plasmonic effects occur primarily when the pattern size is equal to or smaller than the optical wavelength. Especially, well‐arrayed or rationally designed nanostructures with subwavelength sizes can dramatically enhance their usefulness as optical elements by utilizing synchronized plasmonic effects combined with constructive or destructive interference.^[^ ^100^ ^]^ As mentioned above, the nTP technique enables the precise fabrication of these nanostructures using versatile materials and allows their application on various substrates with different optical characteristics. Considering that the practical dimensional resolution of nTP is around 50 nm, this technology finds widespread utility for both visible light (wavelengths of 350–800 nm) applications and for near‐infrared rays (wavelengths of 800–2500 nm) applications. This versatility of nTP facilitates the realization of diverse nanophotonic devices such as plasmonic cut‐off filters,^[^ ^56^ ^]^ holograms,^[^ ^32^, ^58^ ^]^ and polarizers.^[^ ^80^, ^101^ ^]^ With these superior advantages, when one develops or utilizes the nTP technique for fabricating nanophotonic devices, three general requirements should be considered. First, applicable materials in nTP are needed to be discussed. Ag is the best material for plasmonics in nanophotonic devices owing to its lowest optical loss in the visible and near‐infrared spectral ranges. However, Ag can be quickly oxidized, suffers severe losses due to high surface roughness, and is expensive. Thus, Au for superior chemical stability or Al for a reasonable price become alternative solutions and are widely used in nanophotonic devices.^[^ ^31^, ^65^ ^]^ Therefore, an nTP method applicable to these plasmonic materials should be selected. Second, the applicable substrate is also an important parameter. The optical characteristics of the substrate, such as transparency, refractive index, and dielectric constant, can directly affect the device's performance. Thus, high‐quality quartz wafers for rigid devices and transparent polymer films such as PMMA and PET for flexible devices are commonly used to reduce the substrate's effect on the designed nanostructures. Third, for similar reasons, irregularity and opacity of the adhesives or sacrificial layers used in nTP should be considered. Therefore, nTPs based on optically specialized adhesive,^[^ ^102^ ^]^ ultra‐thin SAM,^[^ ^31^ ^]^ or adhesive/sacrificial layer‐free processes^[^ ^4^ ^]^ are preferred in the fabrication of nanophotonic devices. Here, detailed explanations of nanophotonic devices considering these requirements are discussed with some representative examples as follows.

**Figure 8:**
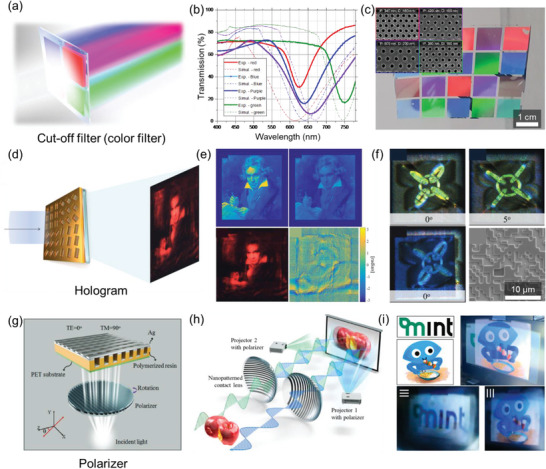
Applications of nTP to nanophotonic devices. a) Schematic illustration of the optical cut‐off filter (color filter). Reproduced with permission.^[^ ^64^ ^]^ Copyright 2022, WILEY‐VCH Verlag GmbH & Co. KGaA, Weinheim. b) Experimental and simulation results of the color filter fabricated by combined nTP processes of adhesive layer‐based *F*_substrate_ strengthening and plasma etching‐based *F*_mold_ weakening. Reproduced with permission.^[^ ^97^ ^]^ Copyright 2023, American Chemical Society. c) Photographic and SEM images of the color filter fabricated by thermoplasticity‐based *F*_substrate_ strengthening strategy. Reproduced with permission.^[^ ^65^ ^]^ Copyright 2022, WILEY‐VCH Verlag GmbH & Co. KGaA, Weinheim. d) Schematic illustration of the hologram. Reproduced under the terms of the CC‐BY‐4.0 license.^[^ ^58^ ^]^ Copyright 2019, The Authors, Published by Springer Nature Limited. e) Experimental and simulation results of the hologram fabricated by adhesive layer‐based *F*_substrate_ strengthening strategy. Reproduced under the terms of the CC‐BY‐4.0 license.^[^ ^58^ ^]^ Copyright 2019, The Authors, Published by Springer Nature Limited. f) Photographic and SEM images of the colorful ring hologram composed of hologram and plasmonic color filter fabricated by thermoplasticity‐based *F*_substrate_ strengthening strategy. Reproduced with permission.^[^ ^32^ ^]^ Copyright 2020, American Chemical Society. g) Schematic illustration of the polarizer. Reproduced with permission.^[^ ^61^ ^]^ Copyright 2017, WILEY‐VCH Verlag GmbH & Co. KGaA, Weinheim. h) Schematic illustration of a wire‐grid polarizer integrated with a soft contact lens fabricated by mold stripping‐based *F*_mold_ weakening strategy. Reproduced with permission.^[^ ^4^ ^]^ Copyright 2021, American Chemical Society. i) Photographic 3D images achieved through binocular parallax‐assisted stereoscopy (i.e., two polarizer lenses with perpendicular orientation). Reproduced with permission.^[^ ^4^ ^]^ Copyright 2021, American Chemical Society.

First, a plasmonic cut‐off filter (e.g., plasmonic color filter) can be effectively fabricated by the nTP technique, as shown in **Figure** **8**a. A plasmonic cut‐off filter is a type of optical filter to selectively transmits or reflects light at specific wavelengths. This is based on the extraordinary optical transmission (EOT), which refers to the phenomenon where the wavelengths of transmitted or reflected light are dependent on the size of well‐arrayed nanostructures (e.g., nanohole array and nanodot array).^[^ ^31^, ^56^, ^59^, ^103^ ^]^ Researchers have generally developed plasmonic color filters using the EOT phenomenon because the color can be easily controlled by changing the size and pitch of the holes or dots in the nanostructures.^[^ ^65^ ^]^ For example, Ahn et al. recently reported a flexible color filter using an Ag nanodot array on a PET substrate.^[^ ^97^ ^]^ In this study, the authors designed the color filter with four colors (red, green, blue, and purple) by finite‐difference time‐domain (FDTD) simulation. Then, the designed nanodot arrays were fabricated by combined nTP processes of adhesive layer‐based *F*_substrate_ strengthening and plasma etching‐based *F*_mold_ weakening. The fabricated colors were well matched with the simulation results obtained in the design process shown in Figure 8b, showing the high reliability of the nTP method. Furthermore, in another study, they developed a flexible color filter using an Ag nanohole array on PMMA substrate fabricated by nTP utilizing thermoplasticity‐based *F*_substrate_ strengthening.^[^ ^65^ ^]^ As shown in Figure 8c, the flexible color filter was successfully realized by designing the hole size and pitch of the nanohole array.

Second, the hologram is one of the most promising applications of nTP because of its unprecedented spatial resolution and large‐area process, as shown in Figure 8d. Precisely fabricated nanostructures by nTP enable the manipulation of the phase and amplitude of light, and it is suitable to the computer‐generated holography (CGH)‐based hologram design method.^[^ ^58^ ^]^ For example, Hwang et al. demonstrated a Beethoven hologram using a CGH‐based design method and nTP utilizing adhesive layer‐based *F*_substrate_ strengthening, as shown in Figure 8e.^[^ ^58^ ^]^ The transferred angular nanoslits composed of Au, Ag, and Al can change the phase of the incident circularly polarized light depending on the orientation angle of the nanoslits, generating the designed holographic interference patterns. Recently, Zhao et al. developed a colorful ring hologram composed of Ag by integrating a hologram and plasmonic color filter,^[^ ^32^ ^]^ as shown in Figure 8f. They claimed that the thermoplasticity‐based *F*_substrate_ strengthening strategy allows double‐faced nTP of two different nanostructures on each side of the PMMA film.

Lastly, nTP is also commonly used to fabricate the plasmonic polarizer, as depicted in Figure 8g. The polarization of the incident light passing a plasmonic polarizer is determined by the direction of the plasmonic wave vector and the direction of the electric field of the incident light. By carefully designing the geometry and arrangement of the metal nanostructures, it is possible to create a polarizer that only transmits light of a specific polarization. The most basic form of polarizer based on the EOT phenomenon is a wire‐grid polarizer, which is composed of a metal nanoline array. This type of polarizer only allows the transmission of light in the transverse magnetic mode.^[^ ^80^, ^101^ ^]^ Recently, Ko et al. developed a wire‐grid polarizer integrated with a soft contact lens, as shown in Figure 8h. They could transfer the Al nanoline array on the curved surface of the soft contact lens through mold stripping‐based *F*_mold_ weakening without any pressure or heat.^[^ ^4^ ^]^ Using two polarizers on the lens with different orientations of nanoline, binocular parallax‐assisted stereoscopy was fabricated, and 3D images were achieved, as shown in Figure 8i. They claimed that the stereoscopic lens fabricated by nTP covers the entire eye, in contrast to existing stereoscopic glasses that suffer from image distortion at their edges, thereby eliminating viewing angle limitations and providing a deep sense of immersion.

In summary, numerous nanophotonic devices have been successfully demonstrated by various nTP methods, verifying the high reliability and versatility of nTP. Therefore, it is anticipated that nTP, which realizes the actual construction of theoretically designed nanostructures with high throughput, will continue to advance and be used extensively.

### 4.3 Chemical and Electrochemical Devices

An increased SA:V ratio is arguably one of the most distinctive characteristics of nanostructures, leading to improved activity in chemical reactions. Therefore, in this section, we will introduce the application of chemical devices that utilize the nTP process for fabricating highly sensitive chemical sensors, as well as designing high‐performance catalysts for energy conversion devices.

**Figure 9:**
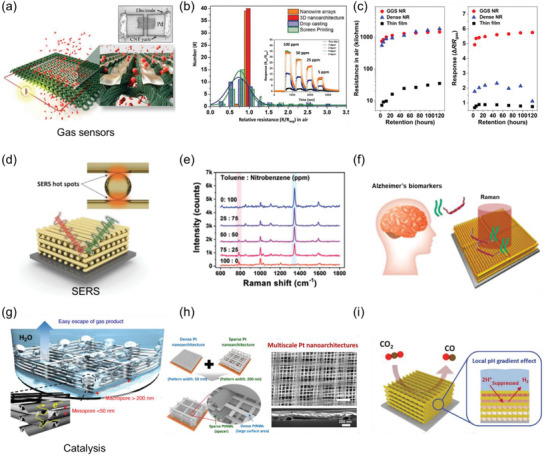
Applications of nTP for fabrication of chemical devices. a) Schematic diagram of the metallic gas sensors fabricated on a textile substrate. Reproduced with permission.^[^ ^15^ ^]^ Copyright 2018, American Chemical Society. b) Distribution of internal resistance of SnO*_x_* gas sensors for various fabrication processes. The inset shows the high sensitivity of the SnO*_x_* nanowire‐based gas sensor. Reproduced with permission.^[^ ^106^ ^]^ Copyright 2022, WILEY‐VCH Verlag GmbH & Co. KGaA, Weinheim. c) The change in the internal resistance of SnO*_x_* nanowires for extended operation at 350 °C. Reproduced under the terms of the CC‐BY‐4.0 license.^[^ ^107^ ^]^ Copyright 2021, The Authors, Published by AAAS. d) Schematic illustration of SERS hot spots at the cross‐point of stacked metallic nanowires. Reproduced with permission.^[^ ^68^ ^]^ Copyright 2016, WILEY‐VCH Verlag GmbH & Co. KGaA, Weinheim. e) Raman spectra of toluene–nitrobenzene mixture gas with various mixing ratios. Reproduced with permission.^[^ ^113^ ^]^ Copyright 2022, WILEY‐VCH Verlag GmbH & Co. KGaA, Weinheim. f) Schematic illustration showing SERS detection of Alzheimer's biomarkers. Reproduced with permission.^[^ ^114^ ^]^ Copyright 2020, American Chemical Society. g) Schematic diagram showing facile bubble transport in 3D stacked catalysts. Reproduced under the terms of the CC‐BY‐4.0 license.^[^ ^118^ ^]^ Copyright 2020, The Authors, Published by Springer Nature Limited. h) Design of hierarchical catalyst structure by nTP. Reproduced under the terms of the CC‐BY‐4.0 license.^[^ ^120^ ^]^ Copyright 2021, The Authors, published by AAAS. i) Schematic diagram of local pH gradient effect in 3D metallic catalysts. Reproduced with permission.^[^ ^119^ ^]^ Copyright 2019, Royal Society of Chemistry.

Gas sensors are devices that can detect trace amounts of gas molecules present in the atmosphere by observing the internal resistance change when exposed to a specific gas (**Figure** **9**a). Among various types of gas sensors, in semiconductor metal oxide‐based gas sensors, oxygen molecules' adsorption and desorption change the depletion layer's width, causing a change in electrical resistance. Therefore, increasing the surface area through nanostructuring would enable highly sensitive detection capabilities of the gas sensor.^[^ ^104^, ^105^ ^]^ However, one of the critical challenges for fabricating nanomaterial‐based gas sensors is the nonuniform and random structures of the chemically synthesized nanomaterials, which leads to poor uniformity and reproducibility of the sensing performance. This issue can be circumvented by fabricating well‐ordered nanowire gas sensor arrays through nTP. For example, Han et al. demonstrated SnO*_x_*‐ and NiO‐based gas sensors for detecting H_2_S and CO gas.^[^ ^106^ ^]^ They utilized the *F*_mold_ weakening‐based nTP process for transferring oxide nanowires on the Si substrate. This enabled systematic analysis of the internal resistance of 3D oxide nanowire arrays as a function of the number of layers to provide insights into the electrical conduction and gas‐sensing response of the 3D nanostructures. While SnO*_x_* nanoparticles prepared by drop casting and screen‐printing show a wide distribution of internal resistance, nanowire arrays and 3D stacked nanowires exhibited a considerably narrow distribution (Figure 9b). The fabricated sensor device could detect H_2_S concentrations of as low as 1 ppm showing a response over *R*_air_/*R*_gas_ = 1200. In addition to the high sensitivity, the nanoscale confinement effect can be another benefit for the stable operation of gas sensors under harsh operating conditions. Han et al. claimed that it is possible to stagnate the grain growth kinetics at high temperatures by synthesizing oxygen‐rich SnO*_x_* nanowires.^[^ ^107^ ^]^ Here, the thickness and corresponding stoichiometry of the nanowire could be controlled during the angled e‐beam deposition before the nTP process. The nanowires were transferred to the Si substrate using S‐nTP, one of the *F*_mold_ weakening‐based nTP processes, to exclude any effects from excessive heat or pressure during the transfer process. Then, they observed the changes in the grain size depending on the annealing time and temperatures. Surprisingly, the grain size did not change after annealing SnO_x_ nanowires at 900 °C for 6 h. The grain growth suppression observed in their work is significant in that the increase in grain size is the main reason for the degraded sensitivity of nanoporous gas sensors. While the SnO_x_ thin film showed a marked increase in the sensor resistance by over 1300% compared to the initial value, oxygen‐rich SnO*_x_* nanowires showed only 180% after 120 h at 350 °C. Several oxide semiconductors and metal‐based gas sensors fabricated by nTP were shown to have high performance, showing the advantages of nTP for developing high‐performance gas sensors.^[^ ^2^, ^17^, ^94^, ^108^ ^]^

Similarly, the sensitivity of surface‐enhanced Raman scattering (SERS) analysis can also be improved by introducing the nTP process, which has emerged as one of the most practical technologies for highly sensitive, label‐free detection of extremely small amounts of molecules. In general, Raman intensity is significantly low for practical use of Raman spectroscopy for detecting chemicals; however, the signal can be amplified up to ≈10^15^ in electromagnetic “hot spots”, where the electromagnetic field is intensified in a small area.^[^ ^109^ ^]^ This results from the phenomenon called localized surface plasmon resonance, a collective oscillation of electrons generated at the interface between the metal surface and the dielectric when the light is irradiated onto the nanostructured metal surface.^[^ ^110^, ^111^ ^]^ Although previous research has focused on decreasing the gap size or increasing the density of hot spots for signal enhancement, the locally distributed and random nature of hot spots inherently caused a uniformity issue, which limits the practical applicability of the SERS analysis. However, 3D metallic nanostructures fabricated by nTP appeared as promising platforms to take advantage of dense hot spots for high sensitivity and evenly distributed nanogaps for high signal uniformity. The schematic shows that 3D stacked metallic nanowires have evenly distributed hot spots formed at the cross‐point between the layers (Figure 9d). Typically, these 3D metallic nanostructures are composed of group 11 metals, such as Cu, Ag, and Au, known to have excellent plasmonic effects.^[^ ^100^ ^]^ Especially, Au has been extensively used as a plasmonic material in SERS substrates due to its high physical and chemical stability, which enables a facile transfer using both *F*_mold_ weakening‐^[^ ^112^ ^]^ or F_substrate_ strengthening‐based nTP.^[^ ^16^, ^67^ ^]^ Jeong et al. found that increasing the number of plasmonic layers leads to stronger signals owing to the formation of denser hot spots.^[^ ^68^ ^]^ However, the signal intensity starts to decrease after stacking more than five layers because the penetration depth of the pump laser is less than hundreds of nanometers. This corroborates that the hot spots formed at the junctions between the topmost and the second layer play the most significant role in signal intensity, which is also confirmed by the finite‐domain time‐difference calculation result. Similarly, Zhao et al. controlled the nanogap size ranging from 1 to 40 nm by adjusting the temperature, pressure, and time above the glass transition temperature of PMMA as the substrate. Using the Rhodamine 6G as a probe molecule, the enhancement factor of two‐layer metal nanostructures was more than five times that in the single‐layer nanostructure, showing the importance of forming nanogap for SERS signal enhancement.^[^ ^16^ ^]^

The aforementioned devices can be combined to realize a chemical sensor with high sensitivity and selectivity. Han et al. showed selective and quantitative estimation of mixed‐gaseous aromatic compounds by designing a multimodal sensor composed of chemo‐resistive and SERS sensors (Figure 9e).^[^ ^113^ ^]^ Conventional single‐component gas sensors have a considerably high sensitivity but cannot differentiate between gases with similar gas adsorption characteristics. On the other hand, SERS analysis provides a unique “fingerprint signal” of the gas molecule, even for molecules with a very similar molecular structure. In this study, quantitative analysis was performed using electrical signals, and qualitative analysis was performed using SERS signals. They fabricated Au nanoparticle‐decorated SnO_2_ nanowire frameworks through nTP of Au‐SnO_2_‐Au structures followed by thermal annealing. Here, Au nanoparticles have a dual functionality of enhancing the response of electrical signals as well as plasmonic particles that amplify Raman signals. The fabricated multimodal sensor detected hazardous chemical gases such as benzene, toluene, and nitrobenzene at the sub‐100 ppm level. In addition, it was shown that the quantification and discrimination of each gas are possible by comparing the normalized intensity of the representative peak of each gas in the gaseous mixture. Cho et al. further improved the selectivity of SERS analyses by introducing functionalized aptamers for quantification to selectively capture target molecules of complex analytes whose composition is unknown.^[^ ^112^ ^]^ It was verified through principal component analysis that the fabricated aptamer‐functionalized monolithic plasmonic nanostructure can accurately quantify bisphenol A, tetracycline, and diclofenac. Park et al. demonstrated the detection of macromolecules such as tau proteins and amyloid β, which are the biomarkers associated with Alzheimer's disease (Figure 9f).^[^ ^114^ ^]^ A carboxylic acid functionalized layer was obtained by forming a graphitic nanolayer through reactive ion etching, which enabled the formation of immobilization sites for protein and uniform coating of the analytes. As a result, accurate quantification of two kinds of polypeptides was possible by the integration of nTP and functionalization processes. SERS‐based analyses can be a potential alternative to conventional analytical methods such as ELISA, NMR, and cryo‐EM, which require complicated analysis or sample preparation procedures and may be applied for the early diagnosis of diseases.

Finally, the nTP technique can be utilized to fabricate high‐performance catalysts for the valorization of carbon products, as well as green and economical hydrogen production. Hydrogen and carbon‐based fuels (e.g., methanol and ethanol) are mainly produced by electrochemical processes such as water electrolysis and CO_2_ reduction reactions. As the electrochemical reaction only occurs in the vicinity of the surface of catalysts, it is crucial to decrease the size of catalysts and increase the surface area to improve the mass activity of the catalysis reaction.^[^ ^72^, ^115^ ^]^ Moreover, retaining the high performance of the catalysts is another critical issue because conventional nanoparticle‐based catalysts experience unavoidable degradation of the performance resulting from the agglomeration of the catalyst particles.^[^ ^116^ ^]^ Therefore, in this section, we will review how the aforementioned issues can be alleviated through 3D nanostructuring realized by the nTP process.

The nTP technique has also been utilized to fabricate 3D‐ordered electrocatalysts for various electrochemical reactions such as oxygen reduction reaction (ORR), oxygen evolution reaction (OER), and CO_2_ reduction reaction (CO_2_RR). One of the main advantages of the 3D‐ordered nanostructure is its ability to facilitate the mass transport of reactants and products.^[^ ^117^ ^]^ Kim et al. fabricated the 3D‐ordered Ir nanostructures using the S‐nTP technique to print Ir nanowires on a Cu foil and subsequently transfer the structure to a Nafion membrane.^[^ ^118^ ^]^ The ordered structure exhibits highly uniform inter‐wire macropores having a period greater than 200 nm. This unique 3D catalyst structure facilitates the elimination of bubbles after temporarily confining them within the well‐defined 3D channels until they reach a critical volume (Figure 9g). The facilitated coalescence of bubbles is followed by easy detachment due to the buoyancy effect, which can overcome the surface tension force between the bubble and catalyst. As a result, a facile detachment of evolved gas bubbles, which is the slowest process in OER, can be achieved through 3D nanostructuring by nTP. The 3D‐ordered nanostructure catalysts not only show an improved catalysis performance through facile delamination of gas bubbles but also exhibit better stability compared to conventional OER catalysts. For example, Ir‐based nanoparticle catalysts are easily degraded and agglomerated after experiencing catalytic reaction cycles due to their extremely small size (typically 2–3 nm diameter) and unstable catalysts–support geometry. However, 3D‐ordered Ir nanostructures are freestanding membranes with interconnected geometry, leading to superior stability with less chance of acidic leaching of Ir.

3D stacking of nanowires can also improve the selectivity of the product of the catalytic reaction. One of the main factors in evaluating the efficiency of the CO_2_RR catalyst is the selectivity of the resulting product. However, the similar redox potential of the hydrogen evolution reaction (HER) to the CO_2_RR can limit the energy conversion efficiency of CO_2_RR due to the formation of hydrogen as a byproduct. The large surface area of the nanostructured catalysts makes it difficult for the byproduct of CO_2_RR (OH^−^) to diffuse and raises the pH level close to the electrode surface, which is called the local pH gradient effect. Cho et al. demonstrated that the insufficient proton concentration on the metal catalysts could hinder the HER, which leads to higher selectivity for desired products (Figure 9h).^[^ ^119^ ^]^ Here, the *F*_mold_ weakening‐based nTP technique was utilized to fabricate 3D stacked nanowires, benefiting the ability to transfer the target material on arbitrary surfaces. Regarding the activity of the catalysts fabricated by nTP, adding more catalyst layers was thought to increase the active sites and improve catalytic performance up to a certain point. However, Kim et al. found that after 20 layers, the improvement reaches its limit due to the mass transfer resistance.^[^ ^120^ ^]^ A master mold with various pattern periods can be used to produce a multiscale nanowire array (NA). The Pt NA was made by alternately stacking dense and sparse nanowire building blocks as a catalyst for ORR. The development of a 3D multiscale Pt NA catalyst with enhanced electrochemical performance compared to that of the 3D ordered Pt nanowires with the dense pattern only has been reported, which was used for Pt NA (Figure 9i). The results showed that the multiscale Pt NA had higher catalytic performances, including improved mass transfer characteristics, especially under high current density conditions.

To summarize, several examples of chemical devices fabricated by the nTP technique were discussed to highlight the beneficial effects of well‐ordered, well‐defined nanostructures, which are distinct from conventional nanoparticles or nanowires with random arrangements, in achieving high‐performance and high‐durability sensors and catalysts. Especially, stacking nanowires by sequential printing using *F*_mold_ weakening‐based nTP can be an effective strategy for applications that require high surface area and structural integrity, as well as ordered nanopores.

## 5 Challenges and Perspectives

Although nTP offers considerable advantages for practical applications as it can produce well‐ordered nanoscale patterns on large areas and at a low cost, the remaining challenges still need further improvement. In this chapter, we will discuss necessary enhancements and potential solutions for nTP, as well as envision the possibilities achievable through advanced nTP technology.

First, challenges arising from physical contact must be addressed. In nTP technology, the transfer of the target material is achieved through contact between the nanopatterned mold and the substrate, resulting in two main issues: defects and alignment. The defect problem, primarily caused by dust particles or contamination, hinders the uniform and reliable transfer of nanopatterns, reducing the overall yield and reliability of the nTP process. While akin to other processes, this issue could be addressed by conducting the entire procedure in a dust‐free cleanroom environment, the associated high costs would undermine the benefits of nTP. Consequently, the development of new materials and processes that facilitate dust‐tolerant nTP is necessary.

Next, the alignment of the nTP process must accompany contact, which is not formed easily with nanoscale accuracy. Therefore, the existing nTP technology has been mainly used for applications that do not require accurate alignment. In earlier chapters, we provided examples of nTP applications, such as the wire grid polarizer, which necessitates only face‐to‐face transfer of single‐layered and periodic nanopatterns, and the multi‐layered, approximately aligned electrochemical catalysts. If nanoscale contact alignment is realized without relying on sophisticated and high‐cost optics or mechanical equipment, it will pave the way for the production of fully designed multi‐material, multi‐junction nanoscale structures. Hence, a substantial increase in the overall maturity and utilization of nTP technology can be anticipated.

Secondly, issues concerning physical vapor deposition (PVD) need to be improved. In conventional nTP, the formation of the target material on the nanomold has been fabricated by PVD with good straightness, such as electron beam evaporation (EBE). Two issues are arising here: limitations in possible film thickness and compatibility for inorganic materials. The ordinarily utilized thickness of films fabricated by methods such as EBE is up to about 1 µm, and it is difficult to use films with a thickness greater than this limitation due to the excessive residual stress.^[^ ^121^ ^]^ Therefore, the current nTP technology is generally utilized for the pattern with lower thickness than that of limitation for the PVD process. In addition, metal has been the most representative candidate for the target material of nTP, but currently, the utilization of inorganic materials such as ceramics nTP is also actively tried. However, inorganic films produced by PVD inevitably have poor physical properties due to their small grain size. Even methods such as EBE, which is the most commonly adopted deposition route, may break the stoichiometry of the compound inorganic materials. Some solutions, such as annealing, have been reported, but they cannot handle the origin of the problem. Hence, the development of a novel deposition method that enables area‐selective deposition of materials while simultaneously ensuring good integrity and even complex stoichiometry is expected to considerably enhance the morphological diversity and physical properties of the transferred patterns.

If the abovementioned issues can be handled, not only the fabrication of hierarchical nano/microscale patterns but also the fabrication of multiple‐layer patterns through the nanoscale alignment and repetitive transfer of each layer. If these improvements can be combined with the various nTP methods introduced in this review, it may provide a novel method to fabricate the nano/micro‐scale 3D structure with heterogeneous materials, the way of fabrication for macro‐scale 3D structure by digital light processing (DLP) type 3D printer. Since the 3D printer provides numerous breakthroughs for various applications, even with the restricted scale (macro‐scale) and materials (light‐curable material), we believe that the future of the nTP technology will provide wider possibilities to diverse research fields since they can provide multi‐scale and multi‐material 3D structuring method.

## 6 Conclusion

In this review, the recent advances in nTP technology and their categorization depending on their working mechanisms based on adhesion force control were discussed. To summarize the overall content, three types of conventional nTP methods (kinetic printing, inking, and stamping, and SAM glue‐based transferring) were introduced in Section 2, and the master mold production methods (KrF lithography and following BCP methods) and their replication methods (casting, thermal molding, UV molding) were discussed in Section 3. Detailed nTP methods (*F*_mold_ weakening, *F*_substrate_ strengthening, and combined method) were also explained in the same chapter. In detail, solvent‐injection‐assisted and mold stripping‐based adhesion weakening methods have been introduced as representatives of *F*_mold_ weakening. Also, adhesive‐based, thermoplasticity‐based, surface tension‐based, and nanowelding‐based adhesion strengthening methods have been introduced as representatives of *F*_substrate_ strengthening. In the case of combined methods, two or more nTP methods are combined for a specific reason: Superimposition of *F*_mold_ weakening has been introduced for reliable large‐area nTP technology with high morphological/material diversity of the target materials and substrates, superimposition of *F*_substrate_ strengthening has been introduced to improve the morphological diversity and mechanical robustness of the transferred nanopattern, and combination of *F*_mold_ weakening and *F*_substrate_ strengthening have been introduced to achieve widespread adoption of nTP and their integration in various applications. A brief overview of these methods can be found in Table 1, and their detailed fabrication conditions can be found in Table 2. Next, various physical/nanophotonic/chemical applications based on these methods were explained in Section 4. Finally, challenges and perspectives about the current nTP technology were discussed in Section 5.

Previous nTP methods have been developed based on a single principle, however, current nTP methods are exploring and improving on these principles and even combining them to suit specific applications. Various methods have been developed for different types of target materials and substrates. These developments have enabled nTP to transfer a wide variety of materials, different scales, and different patterns. As discussed in Chapter 5, the main challenges for further development of nTP lie in the technical limitations of the methods used for nTP, rather than the intrinsic capabilities of nTP itself. As such, it is expected that the technological level of nTP will progress alongside advancements in the technology required for nTP. Therefore, conducting research on the technologies that serve as prerequisites for nTP in parallel with research on nTP technology is believed to be crucial for improving the overall technological level of nTP. As these prerequisite technologies continue to advance, it is expected that nTP technology will become an even more powerful tool for nano/micro‐structuring.

## Conflict of Interest

The authors declare no conflict of interest.
